# Genomic Analysis and Tracking of SARS‐CoV‐2 Variants in Gwangju, South Korea, From 2020 to 2022

**DOI:** 10.1111/irv.13350

**Published:** 2024-06-25

**Authors:** Yeong‐Un Lee, Kwangho Lee, Hongsu Lee, Jung Wook Park, Sun‐Ju Cho, Ji‐Su Park, Jeongeun Mun, Sujung Park, Cheong‐mi Lee, Juhye Lee, Jinjong Seo, Yonghwan Kim, Sun‐Hee Kim, Yoon‐Seok Chung

**Affiliations:** ^1^ Division of Emerging Infectious Disease, Department of Infectious Disease Research Health and Environment Research Institute of Gwangju Gwangju Republic of Korea; ^2^ Division of High‐Risk Pathogens, Bureau of Infectious Diseases Diagnosis Control Korea Disease Control and Prevention Agency (KDCA) Cheongju Republic of Korea

**Keywords:** COVID‐19, lineages, SARS‐CoV‐2, variants, whole‐genome sequencing

## Abstract

**Background:**

Since severe acute respiratory syndrome coronavirus 2 (SARS‐CoV‐2) was first reported in Wuhan, China, in December 2019, it has spread rapidly, and many coronavirus disease (COVID‐19) cases have occurred in Gwangju, South Korea. Viral mutations following the COVID‐19 epidemic have increased interest in the characteristics of epidemics in this region, and pathogen genetic analysis is required for infection control and prevention.

**Methods:**

In this study, SARS‐CoV‐2 whole‐genome analysis was performed on samples from patients with COVID‐19 in Gwangju from 2020 to 2022 to identify the trends in COVID‐19 prevalence and to analyze the phylogenetic relationships of dominant variants. B.41 and B.1.497 prevailed in 2020, the early stage of the COVID‐19 outbreak; then, B.1.619.1 mainly occurred until June 2021. B.1.617.2, classified as sublineages AY.69 and AY.122, occurred continuously from July to December 2021. Since strict measures to strengthen national quarantine management had been implemented in South Korea until this time, the analysis of mutations was also able to infer the epidemiological relationship between infection transmission routes. Since the first identification of the Omicron variant in late December 2021, the spread of infection has been very rapid, and weekly whole‐genome analysis of specimens has enabled us to monitor new Omicron sublineages occurring in Gwangju.

**Conclusions:**

Our study suggests that conducting regional surveillance in addition to nation‐level genomic surveillance will enable more rapid and detailed variant surveillance, which will be helpful in the overall prevention and management of infectious diseases.

## 1 Introduction

Since coronavirus disease (COVID‐19) cases were first reported in Wuhan, Hubei Province, China, in December 2019, severe acute respiratory syndrome coronavirus 2 (SARS‐CoV‐2) has rapidly spread worldwide, and the World Health Organization (WHO) officially declared COVID‐19 a pandemic with the highest alert level on March 11, 2020 [1]. The first confirmed COVID‐19 case in South Korea was identified as a Chinese entry from Wuhan on January 20, 2020. Starting with the first reported case of a Korean patient with COVID‐19 who returned from Thailand and was diagnosed on February 3, 2020, there have been many cases of COVID‐19 infection through imported cases and local outbreaks in Gwangju, South Korea [2, 3].

SARS‐CoV‐2 (the causative agent of COVID‐19) constantly evolves, and although most mutations do not alter viral characteristics, some mutations can affect transmission, pathogenicity, and vaccine and therapeutic efficacy [4]. The WHO monitors the occurrence of significant changes in COVID‐19 variants through amino acid substitution and classifies them into variants of concern (VOCs) and variants of interest, updates them regularly, and recommends public health measures [5].

Viral mutations can be identified through genetic analysis. Whole‐genome sequencing has been widely used recently, and genetic information associated with COVID‐19 variants has been shared worldwide through the Global Initiative on Sharing All Influenza Data (GISAID) and Phylogenetic Assignment of Named Global Outbreak Lineages (PANGOLIN) [6]. Accordingly, we established a whole‐genome analysis in May 2021 to trace the source of infection and confirm genetic mutations in COVID‐19 cases.

As the lineage transitions of SARS‐CoV‐2 increase, several institutions have constructed naming systems to establish standardized nomenclature. GISAID, NextStrain, and PANGOLIN are the most common naming conventions used by scientific communities around the world [7, 8, 9].

The GISAID system is named based on a large‐scale clade defined by the marker variant of the reference genome WIV04 (Genbank: MN996528.1), which is a simple system, but it is inconsistent and classified only by a few mutations. The SARS‐CoV‐2 sequence in GISAID can be easily analyzed using NextStrain, a system that uses phylogenetic analysis to identify evolutionarily stable lineages and sublineages [10]. Once identified, they are named based on their year of appearance and consecutive characters. Specific sublineages are identified as additional information; however, there are no systematic rules. To overcome this problem, the dynamic PANGOLIN system based on evolutionary relations also considers the mechanical relevance of lineages. According to this system, each lineage name consists of an alphabetical prefix and a numeric suffix separated by a period or point. The PANGOLIN system provides detailed and informative outbreak cluster information [6].

In this study, we identified COVID‐19 variants, including VOCs, and performed mutational and phylogenetic analyses of the dominant strain using whole‐genome sequencing of COVID‐19 confirmed cases in Gwangju, Korea, from February 2020 to December 2022. SARS‐CoV‐2 can be continuously monitored to prevent the spread of infectious diseases by tracking new variants that affect pathogenicity, transmissibility, and vaccine efficacy. In addition, a survey of the evolutionary history of mutant viruses will help prepare countermeasures against newly emerging VOCs [11].

## 2 Material and Methods

### 2.1 Sample Collection

Samples were collected using nasopharyngeal and oropharyngeal swabs in universal transport medium between February 2020 and December 2022 from people visiting public centers in five districts of Gwangju Metropolitan City, including suspected patients with COVID‐19, close contacts of confirmed cases, and those seeking testing. Real‐time PCR was performed to detect SARS‐CoV‐2 viral RNA using a PowerChek™ SARS‐CoV‐2 Real‐time PCR Kit (Kogenebiotech, Seoul, South Korea), and samples with high virus copy numbers were selected for sequencing. Most of the samples were randomly selected, while some were chosen based on epidemiological information, focusing on outbreak cases.

### 2.2 Library Preparation and Sequencing

Total RNA was extracted from 140 μL of nasopharyngeal and oropharyngeal swab fluid using a QIAamp Viral RNA Mini Kit (QIAGEN, Hilden, Germany) according to the manufacturer's manual. Libraries were prepared using an Illumina COVIDSeq Assay Kit (Illumina, San Diego, CA, USA) according to the manufacturer's instructions. The RNA was reverse‐transcribed to synthesize cDNA using a random hexamer primer. The cDNA was amplified using two primers based on ARTIC, which cover the entire SARS‐CoV‐2 genome. The amplified PCR product was fragmented, and the PCR amplicons were tagged using the IDT‐ILMN Nextera DNA UD Index Set A. The libraries were pooled, cleaned, and quantified using Qubit™ 1X dsDNA HS (high sensitivity) Assay Kits on a Qubit 3 Fluorometer (Thermo Fisher Scientific, Waltham, MA, USA). The final loading concentration was 9 pM. Sequencing was performed on a MiSeq instrument (Illumina, San Diego, CA, USA) using an Illumina MiSeq Reagent Kit v2 (300 cycles) with dual‐indexed paired‐end 2*151 bp reads.

### 2.3 Sequence Data and Analysis

FASTQ sequencing files were generated from MiSeq and imported to the CLC Genomics Workbench ver. 21.0.3 (CLC bio, QIAGEN, Aarhus, Denmark) to run the workflow. The workflow involved the mapping, alignment, and generation of consensus sequences. The sequence reads were mapped to the SARS‐CoV‐2 reference genome (NCBI: NC045512). Only high‐coverage whole‐genome sequences were used for analysis. The PANGOLIN (https://pangolin.cog‐uk.io/) web application and the NextClade tool (https://clades.nextstrain.org/) were used to identify the SARS‐CoV‐2 lineage.

### 2.4 Phylogenetic Analysis

Phylogenetic analysis was conducted using Molecular Evolutionary Genetics (MEGA‐11 ver. 11.0.13). A phylogenetic tree was created using the maximum likelihood method and Tamura and Nei 1993 (TN93), and the general time reversible model with gamma distribution and invariant sites (G + I) parameters as the bestfit model of nucleotide substitution with 1000 bootstrap replications. FASTQ sequencing files were generated from MiSeq and imported to the CLC Genomics Workbench ver. 21.0.3 (CLC bio, QIAGEN, Aarhus, Denmark) to run the workflow. The workflow involved the mapping, alignment, and generation of consensus sequences. The sequence reads were filtered at or above 30, trimmed for quality, and mapped to the SARS‐CoV‐2 reference genome (NCBI: NC045512). Sequenced samples with ≥ 95% at 20X depth genome coverage were used for analysis. PANGOLIN and NextStrain were used to identify the SARS‐CoV‐2 lineage.

## 3 Results

### 3.1 SARS‐CoV‐2 Cases During Pandemic Waves in Gwangju, South Korea

**FIGURE 1:**
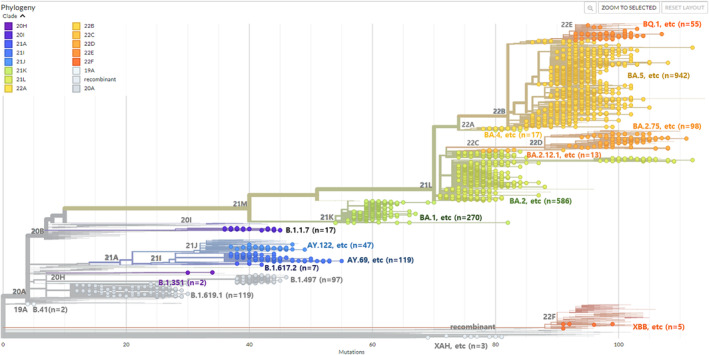
A phylogenetic tree was created using NextClade online software and visualized using the Auspice online tool from metadata produced by NextClade (http://clades.nextstrain.org, accessed on June 30, 2023). A total of 2399 severe acute respiratory syndrome coronavirus 2 (SARS‐CoV‐2) cases detected in Gwangju between February 2020 and December 2022 were uploaded in order to visualize the phylogenetic placement in comparison with published sequences globally. NextStrain clades are broken down according to the indicated color code.

By the end of December 2022, a total of 850,707 cases had been reported in Gwangju since the first COVID‐19 infection in February 2020. When classified by epidemic period, there were 36 cases in the first wave, including the first imported case of COVID‐19 and its occurrence in metropolitan Seoul, Daegu, and Gyeongbuk (February 2020–May 2020). In the second wave, 725 cases were confirmed in a rapid spread throughout the Seoul Metropolitan Area, including outbreaks in assemblies and religious organizations (June 2020–November 2020). During the third wave, when a new COVID‐19 variant appeared and spread nationwide (December 2020–June 2021), 2285 patients were diagnosed with COVID‐19. In addition, 17,123 cases occurred in the fourth wave, spreading the Delta variant (July 2021–January 2022). In the fifth wave, the number of confirmed cases increased explosively nationwide to 518,055 patients due to the Omicron variant, and 312,483 cases were confirmed during the period of the continued emergence of new Omicron variants (July 2022–December 2022) [12]. We randomly selected 2399 COVID‐19 cases by classifying patients with or without a confirmed infection route. Then, whole‐genome sequencing was performed to assign lineages. The sequences identified 147 lineages based on the PANGOLIN criteria (v. 4.3) (Table 1 and Table S1). According to the NextClade classification [13], 16 different clades were detected in Gwangju: 19A, 20A, 20H, 20I, 21A, 21I, 21J, 21K, 21L, 22A, 22B, 22C, 22D, 22E, 22F, and the recombinant strain. Figure 1 shows the phylogenetic tree constructed using the NextClade online tool (accessed on June 30, 2023).

**TABLE 1:** The number of COVID‐19 cases, sequencing cases for representative surveillance, and SARS‐CoV‐2 lineage distribution in Gwajngju from 2020 to 2022.

Period	Number of COVID‐19 cases	Number of sequencing cases	Pango lineage (number of variants)
Total	850,707	2399	2399
1st wave (2020.2. ~ 2020.5.)	36	2	B.41 (2)
2nd wave (2020.6. ~ 2020.11.)	725	35	B.1.497 (35)
3rd wave (2020.12. ~ 2021.6.)	2285	165	B.1.497 (62), B.1.1.7 (15), B.1.351 (1), B.1.619.1 (84), and B.1.617.2 (1)
4th wave (2021.7. ~ 2022.1.)	17,123	312	B.1.1.7 (2), B.1.351 (1), B.1.619.1 (35), B.1.617.2 (168), BA.1 (96), and BA.2 (10)
5th wave (2022.2. ~ 2022.6.)	518,055	756	B.1.617.2 (2), BA.1 (174), BA.2 (504), BA.4 (11), BA.5 (64), and recombinant (1)
6th wave (2022.7.~)	312,483	1129	BA.2 (183), BA.4 (6), BA.5 (933), and recombinant (7)

### 3.2 Dominant SARS‐CoV‐2 Lineages During Waves

**FIGURE 2:**
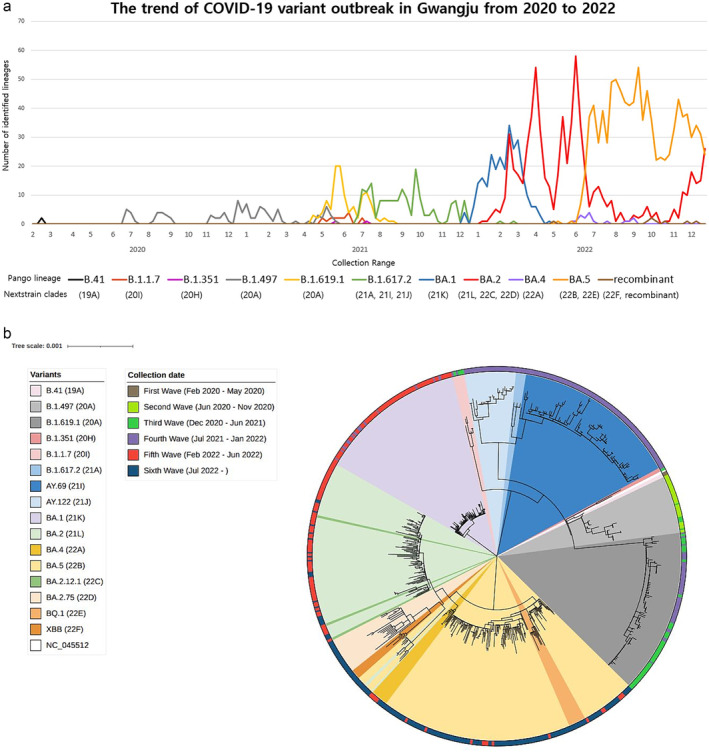
Investigation of SARS‐CoV‐2 variants in Gwangju from 2020 to 2022. (a) The number of identified lineages by month of specimen collection dates from February 2020 to December 2022. A total of 2399 specimens were assigned into 11 Pango lineages and 16 NextStrain clades. (b) Phylogenetic tree showing the relationship between the 596 Gwangju SARS‐CoV‐2 genome sequences. The list of sequences used to create the tree is presented in Table S2. The tree was created with the maximum likelihood method as implemented by MEGA‐11 (ver. 11.0.13) under the general time reversible plus gamma nucleotide substitution model and decorated with iTOL v6. Variants were highlighted in 16 colors. The outer circle–colored strip displays the specimen collection date, sorted from first wave to sixth wave. A Newick (plain text) version of the phylogenetic tree, with branch lengths and support values at nodes, is reported in Supporting Information S1.

Sequences from Gwangju were included along with the global sequences to determine their phylogenetic relationships. Figure 2 shows a phylogenetic tree based on the PANGOLIN classification (Figure 2a) and indicates the epidemic period classified by sample collection date and variants relevant to that period (Figure 2b).

During the first wave, two cases were identified as B.41, and 35 cases were identified as B.1.497 during the second wave. The third wave saw a diverse distribution: B.1.619.1 at 50.9%, B.1.497 at 37.6%, B.1.1.7 at 9.1%, and other variants. During the fourth wave, B.1.617.2 dominated at 53.8%, followed by BA.1 at 30.8% and B.1.619.1 at 11.2%, with Delta becoming predominant. In the fifth wave, Omicron variants were prevalent: BA.2 at 66.6%, BA.1 at 23.0%, and BA.5 at 8.6%. The sixth wave featured a significant presence of Omicron sublineages, with BA.5 at 82.6%, BA.2 at 16.2%, and recombinant variants also confirmed (Figure S1).

### 3.3 B.1.619.1 Genome Sequences

In the early days of the COVID‐19 outbreak, B.1.497 with a D614G mutation in the spike protein and B.1.619.1 with additional mutations, such as N440K and E484K, were prevalent. We focused on the B.1.619.1 variant, which was dominant in Gwangju until the fourth wave when the B.1.617.2 (Delta variant) spread. Among the sequences corresponding to B.1.619.1, the FASTA files of 86 sequences, excluding those with low‐quality data, were used for analysis. We observed 125 nucleotide mutations and 61 amino acid mutations after analyzing the mutation sites and numbers of 86 B.1.619 mutant strains in this study and comparing them with the phylogenetic analysis reference strain (Wuhan‐Hu 1, NC045512) provided by NextClade. Regarding the distribution of amino acid mutations in individual genes, ORF1ab had the highest number of mutations (30), followed by 12 mutations in S, eight in N, five in ORF7a, three in ORF3a, two in ORF8, and one in M.

Among a total of 61 amino acid mutations, 20 mutations were identified in all 86 sequences: eight mutations in ORF1ab (A2123V, E2607L, S3675del, G3676del, F3677del, M3752I, K3929R, and P4715L), seven mutations in S (I210T, N440K, E484K, D614G, D936N, S939F, and T1027I), three mutations in N (P13L, S201I, and T205I), one in M (I82T), and one in ORF7a (E22D) (Table S3).

**FIGURE 3:**
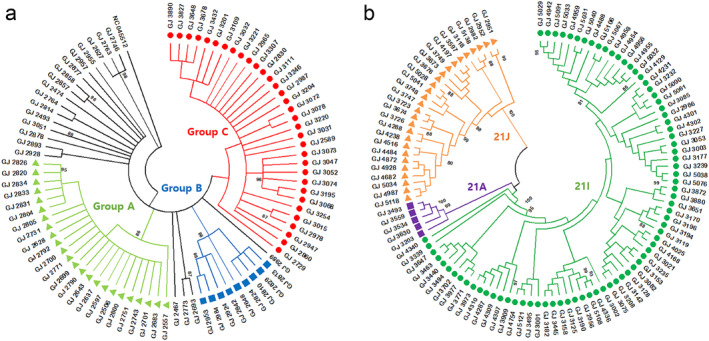
Analysis of relationships among specific variants identified in this study. (a) A maximum likelihood phylogenetic tree of the B.1.619.1 sequence was generated. The sequences were divided into three groups. Group A indicated light green triangles, Group B indicated blue squares, and Group C indicated red circles. (b) A phylogenetic tree of B.1.617.2 sequences was created using the maximum‐likelihood method and the general time reversible model with gamma distribution and invariant site parameters as the bestfit model of nucleotide substitution. Each sequence was applied to the NextStrain online tool, and the divided clades were confirmed. The sequences were divided into three clades: 21A (purple square), 21I (green circle), and 21J (orange triangle).

To analyze the genetic relationship among the 86 B.1.619.1 cases identified in this study, we performed phylogenetic analysis using MEGA‐11 software (Figure 3a). The sequences were grouped into three clusters. Group A comprised a cluster of 24 cases in May and June 2021, including 18 where the route of infection was confirmed. Group B consisted of nine patients who had been in contact with confirmed cases in different regions of Korea. Group C comprised 33 cases detected in July and August 2021, 31 of which had an additional F548S amino acid change in ORF1ab.

### 3.4 B.1.617.2 Genome Sequences

**FIGURE 4:**
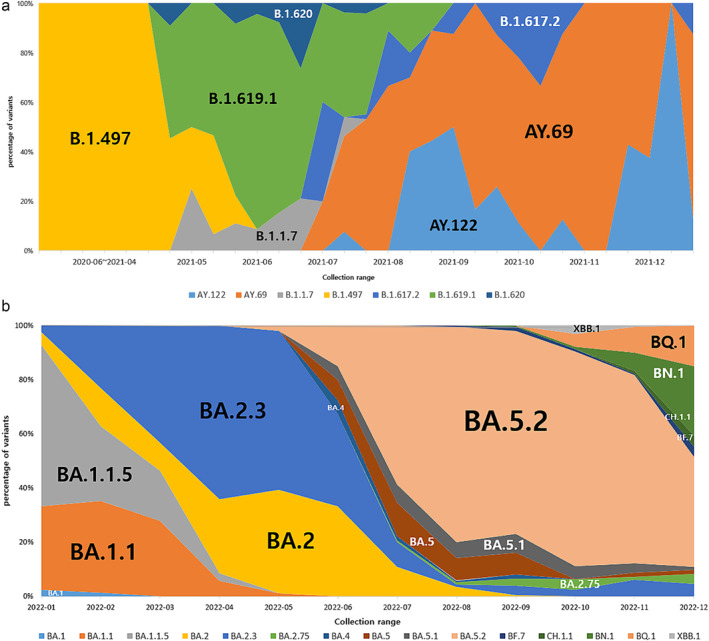
Distribution of COVID‐19 variants by the most prevalent lineages in Gwangju, 2020–2022. (a) Percentage of SARS‐CoV‐2 lineages during the first to fourth epidemic period. Data are shown for lineages B.1.497, B.1.1.7, B.1.619.1, B.1.620, B.1.617.2, AY.69, and AY.122. (b) Percentage of SARS‐CoV‐2 Omicron sublineages during the fifth to sixth epidemic period. A stacked area plot showed the monthly detection rate of various Omicron sublineages.

B.1.619.1 gradually disappeared as B.1.617.2 was introduced in the second half of 2021 and became the dominant variant from July to December 2021 after its confirmation in June 2021. In particular, the sublineages of B.1.617.2, namely, AY.69 and AY.122, accounted for most of the cases (Figure 4a). To analyze the genetic relationships of B.1.617.2 identified in this study, we created a phylogenetic tree with 108 sequences of B.1.617.2 using MEGA‐11 (ver. 11.0.13) software (Figure 3b). Samples collected from COVID‐19 patients in Gwangju were classified into three clades: 21A, 21I, and 21J.

Clade 21A included five samples related to the inflow of the metropolitan area, clade 21J consisted of 27 samples related to imported cases and the enumeration of foreigners, and clade 21I included 76 cases of various infection groups that occurred sporadically in Gwangju. Thus, they were grouped based on the common route of infection and classified into sublineages.

### 3.5 Omicron Variant Spread in Gwangju in 2022

By the end of December 2021, Omicron had spread rapidly against Delta, becoming the dominant variant within several weeks. This result appeared 2 weeks after the first report of Omicron in Gwangju, and its spread was faster than that of the previous variants. As the prevalence of Omicron prolonged, its sublineages continued to appear. Figure 4b shows the distribution of the COVID‐19 Omicron variants in 2022. BA.1 and BA.2 were identified as the dominant variants from January to May 2022. BA.1 was the dominant variant for 10 weeks starting from the 1st week of 2022, and the Omicron sublineages BA.1.1 and BA.1.1.5 accounted for a large proportion. BA.2 was the dominant variant in the 12th and 13th weeks of 2022, and the proportion of sublineage BA.2.3 was also high. Subsequently, both BA.4 and BA.5 were detected, and BA.5 became the dominant variant in June 2022, indicating a tendency for various sublineages to occur simultaneously. BF.7, BQ.1, BQ.1.1, XBB, and XBB.1 were detected since November 2022, and sublineages of BA.2.75, BN.1, and CH.1.1 were confirmed.

## 4 Discussion

We used whole‐genome sequencing to monitor variants that could be new inflows into Gwangju, South Korea, or propagate within the region. Genome analysis of SARS‐CoV‐2 in Gwangju showed that B.1.497 was prevalent in the early days of the COVID‐19 outbreak. Since then, B.1.1.7 (Alpha variant), first reported in the United Kingdom in September 2020, was partially confirmed through infection in the region but did not spread significantly. In addition, all cases of B.1.351 (Beta variant), first reported in South Africa in May 2020, were imported, and there were few cases of local transmission. P.1 (Gamma variant), which was first identified in Brazil in November 2020, was not identified, and mainly B.1.619.1 occurred during this time [14]. In this study, we noted the existence of the B.1.619.1 variant, which is not classified as a VOC by the WHO but was identified as the dominant strain in Gwangju, South Korea, until the introduction of the Delta variant. According to WHO data, the B.1.619 variant was first reported in May 2020, managed as a variant under monitoring on July 14, 2021, and reclassified as a formerly monitored variant on November 9, 2021. In addition, it is difficult to find a reference for the B.1.619 variant, but we confirmed that the mutant virus, which was widespread in Central Africa, spread to Europe using GISAID data [15].

The B.1.619 variant in South Korea was first confirmed in immigrants from Cameroon in February 2021 but was later confirmed to be distinct from those of European countries based on sequence phylogenetic analysis of the B.1.619 variant isolated in Korea. It has been reported that ORF1ab is reclassified as sublineage B.1.619.1 due to the additional presence of the K3929R mutation [16]. Most variants are subdivided according to the processes of propagation and spread to new countries. In this study, as previously reported in South Korea, the B.1.619.1 variant was reclassified as a sublineage of the B.1.619 variant.

We focused on the B.1.619.1 variant because of its mutations, specifically E484K and N440K, which affect the antigenicity of the spike protein. The E484K mutation was identified in both the B.1.351 (Beta variant) and P.1 (Gamma variant) strains, making it an escape mutation that weakens the binding affinity between the neutralizing antibody and the RBD. This reduces antibody effectiveness and is a significant factor in the ability to evade vaccine‐induced antibodies [17, 18]. The N440K mutation increases the affinity for angiotensin‐converting enzyme 2 and confers resistance to monoclonal antibodies [19]. The emergence of variants associated with contagiousness and immune evasion can lead to more cases of reinfection and negatively affect vaccines and therapeutic effects [20, 21]. Notably, common mutations and deletions were observed in the B.1.619.1 and B.1.351 variants in this study, namely, S3675‐3677del and P4715L in ORF1ab, T205I in N, E484K and D614G in S, and S84L in ORF8.

Like the B.1.619.1 variant, AY.69 and AY.122 were identified as the B.1.617.2 (Delta) sublineages that occurred in large numbers in South Korea, including Gwangju. According to previously reported data, AY.69 was specifically distributed in South Korea, and AY.122 was reported to have spread widely in Russia as well [22, 23]. As new mutations occur through intraregional transmission or new variants are introduced from other regions, conducting surveillance in each region along with national genome surveillance will increase the efficiency of infectious disease prevention and management.

During the early stages of the COVID‐19 pandemic, large outbreaks were mostly associated with specific groups. However, the spread of the virus was also closely related to everyday activities, small gatherings, and contact with infected individuals. Accordingly, measures to strengthen quarantine management, such as an order to ban gatherings, were implemented, and an epidemiological investigation was systematically carried out, which we used to identify the route of infection [24]. For patients whose route of infection was unclear, an epidemiological link was inferred through whole‐genome sequencing. Phylogenetic tree analysis of the sequences corresponding to B.1.619.1 and B.1.617.2 (Delta) showed that cases with unidentified infection routes formed a cluster with other cases, and those with confirmed contact with other regions formed a single cluster. COVID‐19 cases in May–June 2021 and July–August 2021 formed different clusters in terms of time. In addition, groups sharing a common route of infection could be classified into the same lineage. These results will provide useful data for tracking the source of infection and blocking the spread of infectious diseases. It is considered necessary to examine the relevance of various mutations to changes in countermeasures against infectious diseases, including vaccination [25, 26].

In the absence of vaccines and treatments in the early stages of the pandemic, strengthened quarantine policies, such as social distancing, were implemented to prevent a surge in the number of COVID‐19 patients responding to SARS‐CoV‐2 [27]. Subsequently, the national vaccination rate reached 70%, and gradual daily recovery increased; however, with mitigated controls and the emergence of the highly contagious Omicron variant, the number of COVID‐19 patients has soared, stopping daily recovery [28]. Due to the rapid increase in confirmed Omicron cases, epidemiological investigation to trace the source of infection was halted, making it virtually meaningless. Thus, owing to the various Omicron sublineages, real‐time reverse transcription PCR targeting some genes could not predict gene mutations and deletion patterns. Therefore, the difficulty in identifying various sublineages was addressed using whole‐genome sequencing [29].

Owing to resource constraints, only selectively analyzed samples were included, thus limiting the representation of the entire case cohort. However, despite this limitation, the analysis enabled the determination of variant distribution and trends within the analyzed subset, offering valuable insights within the scope of the study's conditions.

We monitored mutations in Gwangju that may be newly introduced or propagating in the area via whole‐genome sequencing analysis. We estimated the epidemiological link between patients with COVID‐19 and unknown sources of infection using phylogenetic analysis. Continuous surveillance of SARS‐CoV‐2 variants tracks the influence of new variants on pathogenicity, propagation power, and vaccine efficacy; ultimately, it can be used as evidence to prevent the spread of infectious diseases. Additional research on the evolutionary history of variants will help to prepare countermeasures against new variants in the future [30, 31].

## Author Contributions

**Yeong‐Un Lee:** conceptualization, investigation, methodology, writing–original draft. **Kwangho Lee:** investigation, methodology. **Hongsu Lee:** investigation. **Jung Wook Park:** investigation. **Sun‐Ju Cho:** investigation. **Ji‐Su Park:** investigation. **Jeongeun Mun:** investigation. **Sujung Park:** investigation. **Cheong‐mi Lee:** investigation. **Juhye Lee:** investigation. **Jinjong Seo:** project administration. **Yonghwan Kim:** project administration. **Sun‐Hee Kim:** conceptualization, project administration, writing–review and editing. **Yoon‐Seok Chung:** conceptualization, project administration, writing–review and editing.

## Conflicts of Interest

The authors declare no conflicts of interest.

### Peer Review

The peer review history for this article is available at https://www.webofscience.com/api/gateway/wos/peer‐review/10.1111/irv.13350.

## Supporting information


**Figure S1** The evolution of SARS‐CoV‐2 variants across several waves. It shows the number and detection rate of COVID‐19 variants confirmed from the first wave to the sixth wave.
**Supporting Information S1**. The information about the phylogenetic tree corresponding to Figure 2 is provided in Newick and phyloXML formats.

**Table S1** The specific sublineages and the number of COVID‐19 variants identified in Gwangju from 2020 to 2022.
**Table S2**. Data of 596 human SARS‐CoV‐2 genomes collected from Gwangju were sequenced in this study and submitted to GISAID (accession ID).
**Table S3**. Table of 61 amino acid mutations identified in all 86 sequences of B.1.619.1.

## Data Availability

All sequences from this study have been deposited and shared in GISAID's EpiCov database (https://www.gisaid.org) under accession numbers EPI_ISL_17696287 to 17696403; 17696991 to 17697133; 17697135 to 17697287; 17697289 to 17697437; 17697439 to 17697546; 17698336 to 17698710; 17698712 to 17698775; 17698800 to 17698804; 17698806, 17698809, 17699313, and 17699367 to 17699370; 17699375 to 17699379; 17701835 to 17701951; 17702288 to 17702458; 17702461 to 17702727; 17702761 to 17703100; 17703103 to 17703453; 17703455 to 17703470; and 17719130 to 17719141.
