# Supplementary figures and images for: Genomic Analysis and Tracking of SARS‐CoV‐2 Variants in Gwangju, South Korea, From 2020 to 2022
Source: Influenza Other Respir Viruses. 2024 Jun 25;18(6):e13350. doi: 10.1111/irv.13350 (PMC11196956; doi:10.1111/irv.13350)

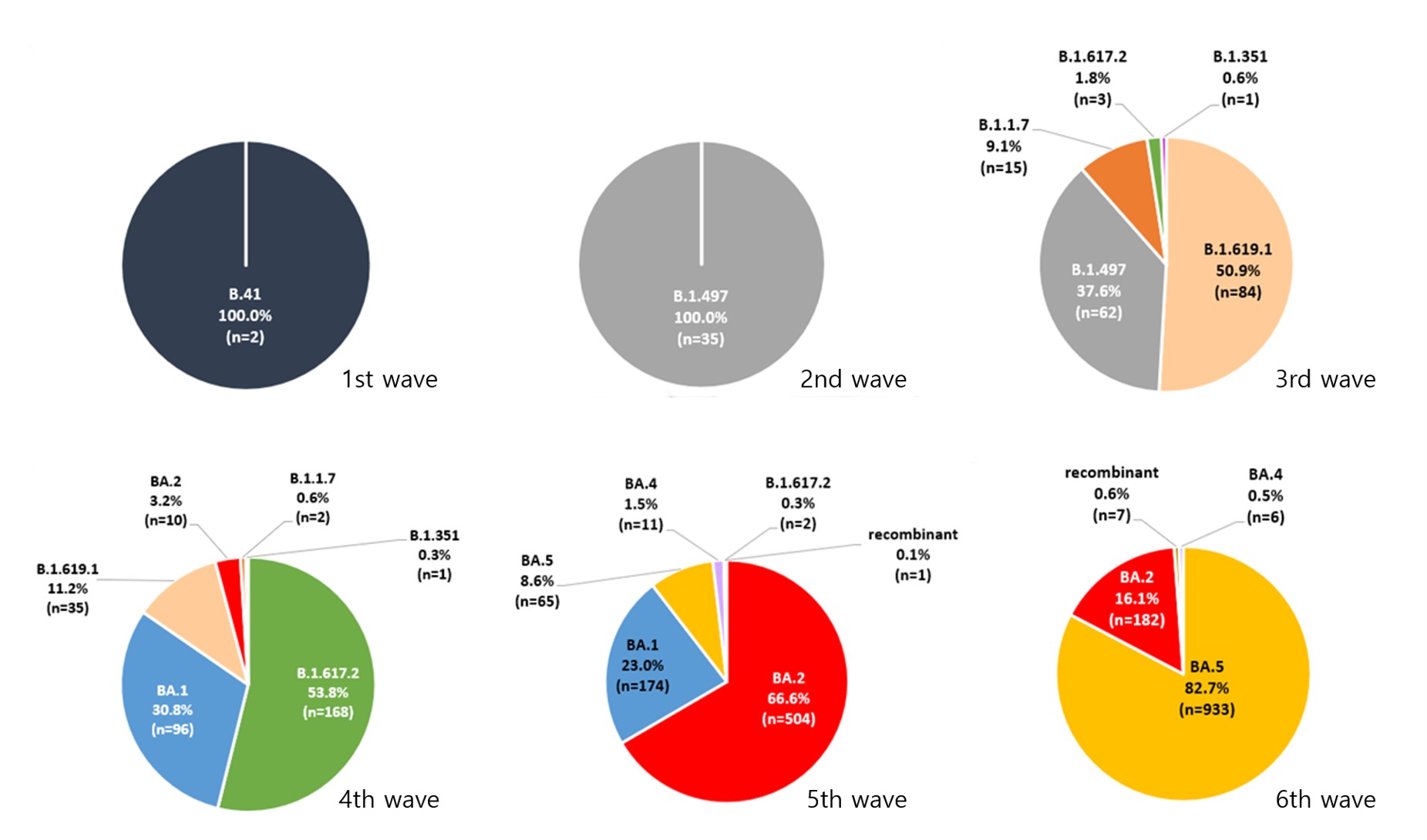

Supplement: Supplementary file 1 — Figure S1 The evolution of SARS‐CoV‐2 variants across several waves. It shows the number and detection rate of COVID‐19 variants confirmed from the first wave to the sixth wave. Supporting Information S1. The information about the phylogenetic tree corresponding to Figure 2 is provided in Newick and phyloXML formats. [file IRV-18-e13350-s001.jpg]
