# Supplementary material for: Genomic Analysis and Tracking of SARS‐CoV‐2 Variants in Gwangju, South Korea, From 2020 to 2022
Source: Influenza Other Respir Viruses. 2024 Jun 25;18(6):e13350. doi: 10.1111/irv.13350 (PMC11196956; doi:10.1111/irv.13350)
Supplement: Supplementary file 2 — Table S1 The specific sublineages and the number of COVID‐19 variants identified in Gwangju from 2020 to 2022. Table S2. Data of 596 human SARS‐CoV‐2 genomes collected from Gwangju were sequenced in this study and submitted to GISAID (accession ID). Table S3. Table of 61 amino acid mutations identified in all 86 sequences of B.1.619.1. [file IRV-18-e13350-s002.docx]

Supplementary Table 1. The specific sublineages and the number of COVID-19 variants identified in Gwangju from 2020 to 2022

| **Pango lineage** | **Total** | **Epidemic period** | **Sublineages (number of variants)** |
| --- | --- | --- | --- |
| B.41 | 2 | 1st wave | B.41 (2) |
| B.1.497 | 97 | 2nd wave | B.1.497 (35) |
|  |  | 3rd wave | B.1.497 (62) |
| B.1.1.7  (alpha) | 17 | 3rd wave  4th wave | B.1.1.7 (15)  B.1.1.7 (2) |
| B.1.351  (Beta) | 2 | 3rd wave | B.1.351 (1) |
|  |  | 4th wave | B.1.351 (1) |
| B.1.619.1 | 119 | 3rd wave | B.1.619.1 (84) |
|  |  | 4th wave | B.1.619.1 (35) |
| B.1.617.2  (Delta) | 173 | 3rd wave | B.1.617.2 (2), AY.69 (1) |
|  |  | 4th wave | B.1.617.2 (5), AY.20 (1), AY.43 (1), AY.69 (115), AY.75.2 (1), AY.106 (1), AY.122 (42), AY.122.5 (2) |
|  |  | 5th wave | AY.69 (1), AY.122.5 (1) |
| BA.1  (Omicron) | 270 | 4th wave | BA.1 (1), BA.1.1 (28), BA.1.1.5 (65), BA.1.15 (1), BA.1.17 (1) |
|  |  | 5th wave | BA.1 (1), BA.1.1 (100), BA.1.1.5 (71), A.1.9 (1), BA.1.17.2 (1) |
| BA.2(Omicron) | 697 | 4th wave | BA.2 (5), BA.2.3 (5) |
|  |  | 5th wave | BA.2 (95), BA.2.1 (1), BA.2.10 (13), BA.2.12.1 (10), BA.2.17 (2), BA.2.3 (286), BA.2.3.1 (1), BA.2.3.10 (1), BA.2.3.12 (12), BA.2.3.13 (4), BA.2.3.14 (11), BA.2.3.2 (4), BA.2.3.8 (4), BA.2.5 (1), BA.2.9 (3), BA.2.40.1 (1), BA.2.51 (1), BA.2.56 (3), BA.2.65 (13), BA.2.68 (38) |
|  |  | 6th wave | BA.2 (14), BA.2.2 (1), BA.2.3 (11), BA.2.3.2 (1), BA.2.3.12 (7), BA.2.3.14 (1), BA.2.3.20 (10), BA.2.3.7 (1), BA.2.12.1 (2), BA.2.18 (1), BA.2.38.1 (2), BA.2.56 (2), BA.2.65 (4), BA.2.68 (1), BA.2.74 (1), BA.2.75 (1), BA.2.75.2 (3), BA.2.75.3 (1), BA.2.75.4 (4), BA.2.75.5 (2), BA.2.76 (1), BG.5 (1), BH.1 (1), BL.1 (7), BM.1.1 (1), BM.1.1.3 (3), BM.4.1.1 (1), BN.1.1 (4), BN.1.2 (15), BN.1.3 (44), BN.1.4 (1), BN.4 (1), BS.1.1 (4), CH.1.1 (10), CM.1 (1), CM.3 (1), CM.4 (13), CM.5 (1), CM.6 (1), CM.8.1 (2) |
| BA.4(Omicron) | 17 | 5th wave | BA.4.1 (5), BA.4.1.1 (6) |
|  |  | 6th wave | BA.4 (1), BA.4.1 (2), BA.4.1.8 (1), BA.4.4 (1), BA.4.6 (1) |
| BA.5(Omicron) | 997 | 5th wave | BA.5.1 (6), BA.5.1.3 (1), BA.5.1.10 (3), BA.5.1.22 (1), BA.5.2 (23), BA.5.2.1 (8), BA.5.2.9 (4), BA.5.5 (10), BA.5.6 (3), BE.1.1 (2), BF.21 (3) |
|  |  | 6th wave | BA.5 (2), BA.5.1 (33), BA.5.1.1 (1), BA.5.1.3 (2), BA.5.1.5 (1), BA.5.1.10 (4), BA.5.1.22 (1), BA.5.1.23 (4), BA.5.1.28 (7), BA.5.2 (365), BA.5.2.1 (217), BA.5.2.2 (3), BA.5.2.3 (2), BA.5.2.6 (10), BA.5.2.9 (1), BA.5.2.12 (1), BA.5.2.16 (2), BA.5.2.19 (23), BA.5.2.20 (14), BA.5.2.22 (12), BA.5.2.25 (1), BA.5.2.26 (3), BA.5.2.27 (9), BA.5.2.28 (3), BA.5.2.32 (4), BA.5.2.34 (3), BA.5.2.44 (2), BA.5.5 (24), BA.5.6 (4), BA.5.6.1 (1), BA.5.8 (1), BA.5.9 (3), BA.5.10 (7), BA.5.10.1 (2), BE.1 (6), BE.1.1 (8), BE.1.1.2 (1), BE.1.4 (1), BE.4 (3), BE.4.1.1 (1), BF.3 (1), BF.5 (43), BF.7 (10), BF.7.4.2 (2), BF.7.5 (1), BF.10 (7), BF.11 (2), BF.21 (8), BF.24 (1), BF.26 (1), BF.27 (1), BF.28 (7), BK.1 (1), BQ.1 (1), BQ.1.1 (19), BQ.1.1.1 (1), BQ.1.1.4 (1), BQ.1.1.17 (7), BQ.1.1.18 (2), BQ.1.2 (9), BQ.1.3 (4), BQ.1.5 (5), BQ.1.11 (4), BQ.1.15 (1), BQ.1.23 (1), CK.3 (1) |
| recombinant | 8 | 5th wave | XAH (1) |
|  |  | 6th wave | XAZ (1), XBB (1), XBB.1 (4), XBC.1 (1) |

Supplementary Table 2. Data of 596 human SARS-CoV-2 genomes collected from Gwangju, sequenced in this study and submitted to GISAID (Accession ID)

| **No.** | **Virus name** | **Accession ID** | **Lineage** | **Label** |
| --- | --- | --- | --- | --- |
| 1 | hCoV-19/South Korea/GJ-HERI-A0030/2020 | EPI_ISL_17696315 | B.41 | 1-1 |
| 2 | hCoV-19/South Korea/GJ-HERI-A0031/2020 | EPI_ISL_17696316 | B.41 | 1-2 |
| 3 | hCoV-19/South Korea/GJ-HERI-A0032/2020 | EPI_ISL_17696317 | B.1.497 | 2-1 |
| 4 | hCoV-19/South Korea/GJ-HERI-A0033/2020 | EPI_ISL_17696318 | B.1.497 | 2-2 |
| 5 | hCoV-19/South Korea/GJ-HERI-A0034/2020 | EPI_ISL_17696319 | B.1.497 | 2-3 |
| 6 | hCoV-19/South Korea/GJ-HERI-A0035/2020 | EPI_ISL_17696320 | B.1.497 | 2-4 |
| 7 | hCoV-19/South Korea/GJ-HERI-A0036/2020 | EPI_ISL_17696321 | B.1.497 | 2-5 |
| 8 | hCoV-19/South Korea/GJ-HERI-A0037/2020 | EPI_ISL_17696322 | B.1.497 | 2-6 |
| 9 | hCoV-19/South Korea/GJ-HERI-A0038/2020 | EPI_ISL_17696323 | B.1.497 | 2-7 |
| 10 | hCoV-19/South Korea/GJ-HERI-A0039/2020 | EPI_ISL_17696324 | B.1.497 | 2-8 |
| 11 | hCoV-19/South Korea/GJ-HERI-A0040/2020 | EPI_ISL_17696325 | B.1.497 | 2-9 |
| 12 | hCoV-19/South Korea/GJ-HERI-A0041/2020 | EPI_ISL_17696326 | B.1.497 | 2-10 |
| 13 | hCoV-19/South Korea/GJ-HERI-A0042/2020 | EPI_ISL_17696327 | B.1.497 | 2-11 |
| 14 | hCoV-19/South Korea/GJ-HERI-A0052/2020 | EPI_ISL_17696337 | B.1.497 | 2-12 |
| 15 | hCoV-19/South Korea/GJ-HERI-A0053/2020 | EPI_ISL_17696338 | B.1.497 | 2-13 |
| 16 | hCoV-19/South Korea/GJ-HERI-A0054/2020 | EPI_ISL_17696339 | B.1.497 | 2-14 |
| 17 | hCoV-19/South Korea/GJ-HERI-A0055/2020 | EPI_ISL_17696340 | B.1.497 | 2-15 |
| 18 | hCoV-19/South Korea/GJ-HERI-A0056/2020 | EPI_ISL_17696341 | B.1.497 | 2-16 |
| 19 | hCoV-19/South Korea/GJ-HERI-A0057/2020 | EPI_ISL_17696342 | B.1.497 | 2-17 |
| 20 | hCoV-19/South Korea/GJ-HERI-A0058/2020 | EPI_ISL_17696343 | B.1.497 | 2-18 |
| 21 | hCoV-19/South Korea/GJ-HERI-A0059/2020 | EPI_ISL_17696344 | B.1.497 | 2-19 |
| 22 | hCoV-19/South Korea/GJ-HERI-A0060/2020 | EPI_ISL_17696345 | B.1.497 | 2-20 |
| 23 | hCoV-19/South Korea/GJ-HERI-A0061/2020 | EPI_ISL_17696346 | B.1.497 | 2-21 |
| 24 | hCoV-19/South Korea/GJ-HERI-A0062/2020 | EPI_ISL_17696347 | B.1.497 | 2-22 |
| 25 | hCoV-19/South Korea/GJ-HERI-A0063/2020 | EPI_ISL_17696348 | B.1.497 | 2-23 |
| 26 | hCoV-19/South Korea/GJ-HERI-A0064/2020 | EPI_ISL_17696349 | B.1.497 | 2-24 |
| 27 | hCoV-19/South Korea/GJ-HERI-A0065/2020 | EPI_ISL_17696350 | B.1.497 | 2-25 |
| 28 | hCoV-19/South Korea/GJ-HERI-A0066/2020 | EPI_ISL_17696351 | B.1.497 | 2-26 |
| 29 | hCoV-19/South Korea/GJ-HERI-A0067/2020 | EPI_ISL_17696352 | B.1.497 | 2-27 |
| 30 | hCoV-19/South Korea/GJ-HERI-A0068/2020 | EPI_ISL_17696353 | B.1.497 | 2-28 |
| 31 | hCoV-19/South Korea/GJ-HERI-A0069/2020 | EPI_ISL_17696354 | B.1.497 | 2-29 |
| 32 | hCoV-19/South Korea/GJ-HERI-A0070/2020 | EPI_ISL_17696355 | B.1.497 | 2-30 |
| 33 | hCoV-19/South Korea/GJ-HERI-A0023/2021 | EPI_ISL_17696308 | B.1.1.7 | 3-1 |
| 34 | hCoV-19/South Korea/GJ-HERI-A0024/2021 | EPI_ISL_17696309 | B.1.1.7 | 3-2 |
| 35 | hCoV-19/South Korea/GJ-HERI-A0025/2021 | EPI_ISL_17696310 | B.1.1.7 | 3-3 |
| 36 | hCoV-19/South Korea/GJ-HERI-A0026/2021 | EPI_ISL_17696311 | B.1.1.7 | 3-4 |
| 37 | hCoV-19/South Korea/GJ-HERI-A0027/2021 | EPI_ISL_17696312 | B.1.1.7 | 3-5 |
| 38 | hCoV-19/South Korea/GJ-HERI-A0029/2021 | EPI_ISL_17696314 | B.1.1.7 | 3-6 |
| 39 | hCoV-19/South Korea/GJ-HERI-A0008/2021 | EPI_ISL_17696294 | B.1.351 | 4-1 |
| 40 | hCoV-19/South Korea/GJ-HERI-A0028/2021 | EPI_ISL_17696313 | B.1.351 | 4-2 |
| 41 | hCoV-19/South Korea/GJ-HERI-A0119/2021 | EPI_ISL_17701835 | B.1.619.1 | 5-1 |
| 42 | hCoV-19/South Korea/GJ-HERI-A0121/2021 | EPI_ISL_17701836 | B.1.619.1 | 5-2 |
| 43 | hCoV-19/South Korea/GJ-HERI-A0122/2021 | EPI_ISL_17701837 | B.1.619.1 | 5-3 |
| 44 | hCoV-19/South Korea/GJ-HERI-A0124/2021 | EPI_ISL_17701839 | B.1.619.1 | 5-4 |
| 45 | hCoV-19/South Korea/GJ-HERI-A0129/2021 | EPI_ISL_17701844 | B.1.619.1 | 5-5 |
| 46 | hCoV-19/South Korea/GJ-HERI-A0130/2021 | EPI_ISL_17701845 | B.1.619.1 | 5-6 |
| 47 | hCoV-19/South Korea/GJ-HERI-A0131/2021 | EPI_ISL_17701846 | B.1.619.1 | 5-7 |
| 48 | hCoV-19/South Korea/GJ-HERI-A0132/2021 | EPI_ISL_17701847 | B.1.619.1 | 5-8 |
| 49 | hCoV-19/South Korea/GJ-HERI-A0133/2021 | EPI_ISL_17701848 | B.1.619.1 | 5-9 |
| 50 | hCoV-19/South Korea/GJ-HERI-A0134/2021 | EPI_ISL_17701849 | B.1.619.1 | 5-10 |
| 51 | hCoV-19/South Korea/GJ-HERI-A0135/2021 | EPI_ISL_17701850 | B.1.619.1 | 5-11 |
| 52 | hCoV-19/South Korea/GJ-HERI-A0136/2021 | EPI_ISL_17701851 | B.1.619.1 | 5-12 |
| 53 | hCoV-19/South Korea/GJ-HERI-A0137/2021 | EPI_ISL_17701852 | B.1.619.1 | 5-13 |
| 54 | hCoV-19/South Korea/GJ-HERI-A0138/2021 | EPI_ISL_17701853 | B.1.619.1 | 5-14 |
| 55 | hCoV-19/South Korea/GJ-HERI-A0139/2021 | EPI_ISL_17701854 | B.1.619.1 | 5-15 |
| 56 | hCoV-19/South Korea/GJ-HERI-A0140/2021 | EPI_ISL_17701855 | B.1.619.1 | 5-16 |
| 57 | hCoV-19/South Korea/GJ-HERI-A0141/2021 | EPI_ISL_17701856 | B.1.619.1 | 5-17 |
| 58 | hCoV-19/South Korea/GJ-HERI-A0142/2021 | EPI_ISL_17701857 | B.1.619.1 | 5-18 |
| 59 | hCoV-19/South Korea/GJ-HERI-A0144/2021 | EPI_ISL_17701859 | B.1.619.1 | 5-19 |
| 60 | hCoV-19/South Korea/GJ-HERI-A0145/2021 | EPI_ISL_17701860 | B.1.619.1 | 5-20 |
| 61 | hCoV-19/South Korea/GJ-HERI-A0146/2021 | EPI_ISL_17701861 | B.1.619.1 | 5-21 |
| 62 | hCoV-19/South Korea/GJ-HERI-A0148/2021 | EPI_ISL_17701863 | B.1.619.1 | 5-22 |
| 63 | hCoV-19/South Korea/GJ-HERI-A0149/2021 | EPI_ISL_17701864 | B.1.619.1 | 5-23 |
| 64 | hCoV-19/South Korea/GJ-HERI-A0150/2021 | EPI_ISL_17701865 | B.1.619.1 | 5-24 |
| 65 | hCoV-19/South Korea/GJ-HERI-A0153/2021 | EPI_ISL_17701868 | B.1.619.1 | 5-25 |
| 66 | hCoV-19/South Korea/GJ-HERI-A0154/2021 | EPI_ISL_17701869 | B.1.619.1 | 5-26 |
| 67 | hCoV-19/South Korea/GJ-HERI-A0156/2021 | EPI_ISL_17701870 | B.1.619.1 | 5-27 |
| 68 | hCoV-19/South Korea/GJ-HERI-A0158/2021 | EPI_ISL_17701872 | B.1.619.1 | 5-28 |
| 69 | hCoV-19/South Korea/GJ-HERI-A0160/2021 | EPI_ISL_17701874 | B.1.619.1 | 5-29 |
| 70 | hCoV-19/South Korea/GJ-HERI-A0161/2021 | EPI_ISL_17701875 | B.1.619.1 | 5-30 |
| 71 | hCoV-19/South Korea/GJ-HERI-A0162/2021 | EPI_ISL_17701876 | B.1.619.1 | 5-31 |
| 72 | hCoV-19/South Korea/GJ-HERI-A0163/2021 | EPI_ISL_17701877 | B.1.619.1 | 5-32 |
| 73 | hCoV-19/South Korea/GJ-HERI-A0164/2021 | EPI_ISL_17701878 | B.1.619.1 | 5-33 |
| 74 | hCoV-19/South Korea/GJ-HERI-A0165/2021 | EPI_ISL_17701879 | B.1.619.1 | 5-34 |
| 75 | hCoV-19/South Korea/GJ-HERI-A0167/2021 | EPI_ISL_17701881 | B.1.619.1 | 5-35 |
| 76 | hCoV-19/South Korea/GJ-HERI-A0168/2021 | EPI_ISL_17701882 | B.1.619.1 | 5-36 |
| 77 | hCoV-19/South Korea/GJ-HERI-A0170/2021 | EPI_ISL_17701884 | B.1.619.1 | 5-37 |
| 78 | hCoV-19/South Korea/GJ-HERI-A0176/2021 | EPI_ISL_17701890 | B.1.619.1 | 5-38 |
| 79 | hCoV-19/South Korea/GJ-HERI-A0178/2021 | EPI_ISL_17701892 | B.1.619.1 | 5-39 |
| 80 | hCoV-19/South Korea/GJ-HERI-A0179/2021 | EPI_ISL_17701893 | B.1.619.1 | 5-40 |
| 81 | hCoV-19/South Korea/GJ-HERI-A0180/2021 | EPI_ISL_17701894 | B.1.619.1 | 5-41 |
| 82 | hCoV-19/South Korea/GJ-HERI-A0183/2021 | EPI_ISL_17701897 | B.1.619.1 | 5-42 |
| 83 | hCoV-19/South Korea/GJ-HERI-A0184/2021 | EPI_ISL_17701898 | B.1.619.1 | 5-43 |
| 84 | hCoV-19/South Korea/GJ-HERI-A0186/2021 | EPI_ISL_17701900 | B.1.619.1 | 5-44 |
| 85 | hCoV-19/South Korea/GJ-HERI-A0187/2021 | EPI_ISL_17701901 | B.1.619.1 | 5-45 |
| 86 | hCoV-19/South Korea/GJ-HERI-A0188/2021 | EPI_ISL_17701902 | B.1.619.1 | 5-46 |
| 87 | hCoV-19/South Korea/GJ-HERI-A0190/2021 | EPI_ISL_17701904 | B.1.619.1 | 5-47 |
| 88 | hCoV-19/South Korea/GJ-HERI-A0191/2021 | EPI_ISL_17701905 | B.1.619.1 | 5-48 |
| 89 | hCoV-19/South Korea/GJ-HERI-A0192/2021 | EPI_ISL_17701906 | B.1.619.1 | 5-49 |
| 90 | hCoV-19/South Korea/GJ-HERI-A0193/2021 | EPI_ISL_17701907 | B.1.619.1 | 5-50 |
| 91 | hCoV-19/South Korea/GJ-HERI-A0194/2021 | EPI_ISL_17701908 | B.1.619.1 | 5-51 |
| 92 | hCoV-19/South Korea/GJ-HERI-A0195/2021 | EPI_ISL_17701909 | B.1.619.1 | 5-52 |
| 93 | hCoV-19/South Korea/GJ-HERI-A0196/2021 | EPI_ISL_17701910 | B.1.619.1 | 5-53 |
| 94 | hCoV-19/South Korea/GJ-HERI-A0197/2021 | EPI_ISL_17701911 | B.1.619.1 | 5-54 |
| 95 | hCoV-19/South Korea/GJ-HERI-A0198/2021 | EPI_ISL_17701912 | B.1.619.1 | 5-55 |
| 96 | hCoV-19/South Korea/GJ-HERI-A0199/2021 | EPI_ISL_17701913 | B.1.619.1 | 5-56 |
| 97 | hCoV-19/South Korea/GJ-HERI-A0200/2021 | EPI_ISL_17701914 | B.1.619.1 | 5-57 |
| 98 | hCoV-19/South Korea/GJ-HERI-A0201/2021 | EPI_ISL_17701915 | B.1.619.1 | 5-58 |
| 99 | hCoV-19/South Korea/GJ-HERI-A0202/2021 | EPI_ISL_17701916 | B.1.619.1 | 5-59 |
| 100 | hCoV-19/South Korea/GJ-HERI-A0203/2021 | EPI_ISL_17701917 | B.1.619.1 | 5-60 |
| 101 | hCoV-19/South Korea/GJ-HERI-A0204/2021 | EPI_ISL_17701918 | B.1.619.1 | 5-61 |
| 102 | hCoV-19/South Korea/GJ-HERI-A0205/2021 | EPI_ISL_17701919 | B.1.619.1 | 5-62 |
| 103 | hCoV-19/South Korea/GJ-HERI-A0206/2021 | EPI_ISL_17701920 | B.1.619.1 | 5-63 |
| 104 | hCoV-19/South Korea/GJ-HERI-A0207/2021 | EPI_ISL_17701921 | B.1.619.1 | 5-64 |
| 105 | hCoV-19/South Korea/GJ-HERI-A0208/2021 | EPI_ISL_17701922 | B.1.619.1 | 5-65 |
| 106 | hCoV-19/South Korea/GJ-HERI-A0209/2021 | EPI_ISL_17701923 | B.1.619.1 | 5-66 |
| 107 | hCoV-19/South Korea/GJ-HERI-A0210/2021 | EPI_ISL_17701924 | B.1.619.1 | 5-67 |
| 108 | hCoV-19/South Korea/GJ-HERI-A0211/2021 | EPI_ISL_17701925 | B.1.619.1 | 5-68 |
| 109 | hCoV-19/South Korea/GJ-HERI-A0212/2021 | EPI_ISL_17701926 | B.1.619.1 | 5-69 |
| 110 | hCoV-19/South Korea/GJ-HERI-A0213/2021 | EPI_ISL_17701927 | B.1.619.1 | 5-70 |
| 111 | hCoV-19/South Korea/GJ-HERI-A0214/2021 | EPI_ISL_17701928 | B.1.619.1 | 5-71 |
| 112 | hCoV-19/South Korea/GJ-HERI-A0215/2021 | EPI_ISL_17701929 | B.1.619.1 | 5-72 |
| 113 | hCoV-19/South Korea/GJ-HERI-A0216/2021 | EPI_ISL_17701930 | B.1.619.1 | 5-73 |
| 114 | hCoV-19/South Korea/GJ-HERI-A0218/2021 | EPI_ISL_17701932 | B.1.619.1 | 5-74 |
| 115 | hCoV-19/South Korea/GJ-HERI-A0219/2021 | EPI_ISL_17701933 | B.1.619.1 | 5-75 |
| 116 | hCoV-19/South Korea/GJ-HERI-A0220/2021 | EPI_ISL_17701934 | B.1.619.1 | 5-76 |
| 117 | hCoV-19/South Korea/GJ-HERI-A0221/2021 | EPI_ISL_17701935 | B.1.619.1 | 5-77 |
| 118 | hCoV-19/South Korea/GJ-HERI-A0222/2021 | EPI_ISL_17701936 | B.1.619.1 | 5-78 |
| 119 | hCoV-19/South Korea/GJ-HERI-A0223/2021 | EPI_ISL_17701937 | B.1.619.1 | 5-79 |
| 120 | hCoV-19/South Korea/GJ-HERI-A0224/2021 | EPI_ISL_17701938 | B.1.619.1 | 5-80 |
| 121 | hCoV-19/South Korea/GJ-HERI-A0225/2021 | EPI_ISL_17701939 | B.1.619.1 | 5-81 |
| 122 | hCoV-19/South Korea/GJ-HERI-A0226/2021 | EPI_ISL_17701940 | B.1.619.1 | 5-82 |
| 123 | hCoV-19/South Korea/GJ-HERI-A0228/2021 | EPI_ISL_17701942 | B.1.619.1 | 5-83 |
| 124 | hCoV-19/South Korea/GJ-HERI-A0229/2021 | EPI_ISL_17701943 | B.1.619.1 | 5-84 |
| 125 | hCoV-19/South Korea/GJ-HERI-A0230/2021 | EPI_ISL_17701944 | B.1.619.1 | 5-85 |
| 126 | hCoV-19/South Korea/GJ-HERI-A0231/2021 | EPI_ISL_17701945 | B.1.619.1 | 5-86 |
| 127 | hCoV-19/South Korea/GJ-HERI-A0238/2021 | EPI_ISL_17702288 | B.1.617.2 | 6-1 |
| 128 | hCoV-19/South Korea/GJ-HERI-A0239/2021 | EPI_ISL_17702289 | B.1.617.2 | 6-2 |
| 129 | hCoV-19/South Korea/GJ-HERI-A0245/2021 | EPI_ISL_17702295 | AY.122 | 6-3 |
| 130 | hCoV-19/South Korea/GJ-HERI-A0246/2021 | EPI_ISL_17702296 | AY.69 | 6-4 |
| 131 | hCoV-19/South Korea/GJ-HERI-A0247/2021 | EPI_ISL_17702297 | AY.69 | 6-5 |
| 132 | hCoV-19/South Korea/GJ-HERI-A0248/2021 | EPI_ISL_17702298 | AY.69 | 6-6 |
| 133 | hCoV-19/South Korea/GJ-HERI-A0249/2021 | EPI_ISL_17702299 | AY.69 | 6-7 |
| 134 | hCoV-19/South Korea/GJ-HERI-A0250/2021 | EPI_ISL_17702300 | AY.69 | 6-8 |
| 135 | hCoV-19/South Korea/GJ-HERI-A0252/2021 | EPI_ISL_17702302 | AY.69 | 6-9 |
| 136 | hCoV-19/South Korea/GJ-HERI-A0253/2021 | EPI_ISL_17702303 | AY.69 | 6-10 |
| 137 | hCoV-19/South Korea/GJ-HERI-A0254/2021 | EPI_ISL_17702304 | AY.69 | 6-11 |
| 138 | hCoV-19/South Korea/GJ-HERI-A0255/2021 | EPI_ISL_17702305 | AY.69 | 6-12 |
| 139 | hCoV-19/South Korea/GJ-HERI-A0256/2021 | EPI_ISL_17719138 | AY.69 | 6-13 |
| 140 | hCoV-19/South Korea/GJ-HERI-A0257/2021 | EPI_ISL_17702306 | AY.69 | 6-14 |
| 141 | hCoV-19/South Korea/GJ-HERI-A0258/2021 | EPI_ISL_17702307 | AY.69 | 6-15 |
| 142 | hCoV-19/South Korea/GJ-HERI-A0259/2021 | EPI_ISL_17702308 | AY.69 | 6-16 |
| 143 | hCoV-19/South Korea/GJ-HERI-A0260/2021 | EPI_ISL_17702309 | AY.69 | 6-17 |
| 144 | hCoV-19/South Korea/GJ-HERI-A0262/2021 | EPI_ISL_17702311 | AY.69 | 6-18 |
| 145 | hCoV-19/South Korea/GJ-HERI-A0263/2021 | EPI_ISL_17719139 | AY.69 | 6-19 |
| 146 | hCoV-19/South Korea/GJ-HERI-A0264/2021 | EPI_ISL_17702312 | AY.69 | 6-20 |
| 147 | hCoV-19/South Korea/GJ-HERI-A0265/2021 | EPI_ISL_17702313 | AY.69 | 6-21 |
| 148 | hCoV-19/South Korea/GJ-HERI-A0266/2021 | EPI_ISL_17702314 | AY.69 | 6-22 |
| 149 | hCoV-19/South Korea/GJ-HERI-A0267/2021 | EPI_ISL_17702315 | AY.69 | 6-23 |
| 150 | hCoV-19/South Korea/GJ-HERI-A0268/2021 | EPI_ISL_17702316 | B.1.617.2 | 6-24 |
| 151 | hCoV-19/South Korea/GJ-HERI-A0269/2021 | EPI_ISL_17702317 | AY.69 | 6-25 |
| 152 | hCoV-19/South Korea/GJ-HERI-A0270/2021 | EPI_ISL_17702318 | AY.69 | 6-26 |
| 153 | hCoV-19/South Korea/GJ-HERI-A0273/2021 | EPI_ISL_17702321 | AY.69 | 6-27 |
| 154 | hCoV-19/South Korea/GJ-HERI-A0274/2021 | EPI_ISL_17702322 | AY.69 | 6-28 |
| 155 | hCoV-19/South Korea/GJ-HERI-A0275/2021 | EPI_ISL_17702323 | AY.122 | 6-29 |
| 156 | hCoV-19/South Korea/GJ-HERI-A0276/2021 | EPI_ISL_17702324 | AY.69 | 6-30 |
| 157 | hCoV-19/South Korea/GJ-HERI-A0277/2021 | EPI_ISL_17702325 | AY.69 | 6-31 |
| 158 | hCoV-19/South Korea/GJ-HERI-A0278/2021 | EPI_ISL_17702326 | AY.69 | 6-32 |
| 159 | hCoV-19/South Korea/GJ-HERI-A0279/2021 | EPI_ISL_17702327 | AY.69 | 6-33 |
| 160 | hCoV-19/South Korea/GJ-HERI-A0280/2021 | EPI_ISL_17702328 | AY.69 | 6-34 |
| 161 | hCoV-19/South Korea/GJ-HERI-A0281/2021 | EPI_ISL_17702329 | AY.69 | 6-35 |
| 162 | hCoV-19/South Korea/GJ-HERI-A0282/2021 | EPI_ISL_17702330 | AY.69 | 6-36 |
| 163 | hCoV-19/South Korea/GJ-HERI-A0283/2021 | EPI_ISL_17702331 | B.1.617.2 | 6-37 |
| 164 | hCoV-19/South Korea/GJ-HERI-A0284/2021 | EPI_ISL_17702332 | AY.69 | 6-38 |
| 165 | hCoV-19/South Korea/GJ-HERI-A0286/2021 | EPI_ISL_17702334 | AY.69 | 6-39 |
| 166 | hCoV-19/South Korea/GJ-HERI-A0287/2021 | EPI_ISL_17702335 | AY.69 | 6-40 |
| 167 | hCoV-19/South Korea/GJ-HERI-A0288/2021 | EPI_ISL_17702336 | AY.69 | 6-41 |
| 168 | hCoV-19/South Korea/GJ-HERI-A0289/2021 | EPI_ISL_17702337 | AY.69 | 6-42 |
| 169 | hCoV-19/South Korea/GJ-HERI-A0293/2021 | EPI_ISL_17702341 | B.1.617.2 | 6-43 |
| 170 | hCoV-19/South Korea/GJ-HERI-A0294/2021 | EPI_ISL_17702342 | B.1.617.2 | 6-44 |
| 171 | hCoV-19/South Korea/GJ-HERI-A0295/2021 | EPI_ISL_17702343 | B.1.617.2 | 6-45 |
| 172 | hCoV-19/South Korea/GJ-HERI-A0296/2021 | EPI_ISL_17702344 | AY.69 | 6-46 |
| 173 | hCoV-19/South Korea/GJ-HERI-A0297/2021 | EPI_ISL_17702345 | AY.122 | 6-47 |
| 174 | hCoV-19/South Korea/GJ-HERI-A0298/2021 | EPI_ISL_17702346 | AY.122 | 6-48 |
| 175 | hCoV-19/South Korea/GJ-HERI-A0299/2021 | EPI_ISL_17702347 | AY.69 | 6-49 |
| 176 | hCoV-19/South Korea/GJ-HERI-A0300/2021 | EPI_ISL_17702348 | AY.122 | 6-50 |
| 177 | hCoV-19/South Korea/GJ-HERI-A0301/2021 | EPI_ISL_17702349 | AY.122 | 6-51 |
| 178 | hCoV-19/South Korea/GJ-HERI-A0302/2021 | EPI_ISL_17702350 | AY.122 | 6-52 |
| 179 | hCoV-19/South Korea/GJ-HERI-A0303/2021 | EPI_ISL_17702351 | AY.122 | 6-53 |
| 180 | hCoV-19/South Korea/GJ-HERI-A0304/2021 | EPI_ISL_17702352 | AY.122 | 6-54 |
| 181 | hCoV-19/South Korea/GJ-HERI-A0305/2021 | EPI_ISL_17702353 | AY.69 | 6-55 |
| 182 | hCoV-19/South Korea/GJ-HERI-A0306/2021 | EPI_ISL_17702354 | AY.69 | 6-56 |
| 183 | hCoV-19/South Korea/GJ-HERI-A0307/2021 | EPI_ISL_17702355 | AY.69 | 6-57 |
| 184 | hCoV-19/South Korea/GJ-HERI-A0308/2021 | EPI_ISL_17702356 | AY.69 | 6-58 |
| 185 | hCoV-19/South Korea/GJ-HERI-A0309/2021 | EPI_ISL_17702357 | AY.122 | 6-59 |
| 186 | hCoV-19/South Korea/GJ-HERI-A0310/2021 | EPI_ISL_17702358 | AY.122 | 6-60 |
| 187 | hCoV-19/South Korea/GJ-HERI-A0311/2021 | EPI_ISL_17702359 | AY.69 | 6-61 |
| 188 | hCoV-19/South Korea/GJ-HERI-A0312/2021 | EPI_ISL_17702360 | AY.69 | 6-62 |
| 189 | hCoV-19/South Korea/GJ-HERI-A0313/2021 | EPI_ISL_17702361 | AY.69 | 6-63 |
| 190 | hCoV-19/South Korea/GJ-HERI-A0314/2021 | EPI_ISL_17702362 | AY.69 | 6-64 |
| 191 | hCoV-19/South Korea/GJ-HERI-A0315/2021 | EPI_ISL_17702363 | AY.69 | 6-65 |
| 192 | hCoV-19/South Korea/GJ-HERI-A0316/2021 | EPI_ISL_17702364 | AY.69 | 6-66 |
| 193 | hCoV-19/South Korea/GJ-HERI-A0317/2021 | EPI_ISL_17702365 | AY.69 | 6-67 |
| 194 | hCoV-19/South Korea/GJ-HERI-A0318/2021 | EPI_ISL_17702366 | AY.69 | 6-68 |
| 195 | hCoV-19/South Korea/GJ-HERI-A0319/2021 | EPI_ISL_17702367 | AY.122 | 6-69 |
| 196 | hCoV-19/South Korea/GJ-HERI-A0320/2021 | EPI_ISL_17702368 | AY.69 | 6-70 |
| 197 | hCoV-19/South Korea/GJ-HERI-A0321/2021 | EPI_ISL_17702369 | AY.122 | 6-71 |
| 198 | hCoV-19/South Korea/GJ-HERI-A0322/2021 | EPI_ISL_17702370 | AY.69 | 6-72 |
| 199 | hCoV-19/South Korea/GJ-HERI-A0323/2021 | EPI_ISL_17702371 | AY.122 | 6-73 |
| 200 | hCoV-19/South Korea/GJ-HERI-A0328/2021 | EPI_ISL_17702376 | AY.122 | 6-74 |
| 201 | hCoV-19/South Korea/GJ-HERI-A0332/2021 | EPI_ISL_17702380 | AY.69 | 6-75 |
| 202 | hCoV-19/South Korea/GJ-HERI-A0334/2021 | EPI_ISL_17702382 | AY.69 | 6-76 |
| 203 | hCoV-19/South Korea/GJ-HERI-A0337/2021 | EPI_ISL_17702385 | AY.69 | 6-77 |
| 204 | hCoV-19/South Korea/GJ-HERI-A0338/2021 | EPI_ISL_17702386 | AY.122 | 6-78 |
| 205 | hCoV-19/South Korea/GJ-HERI-A0339/2021 | EPI_ISL_17702387 | AY.69 | 6-79 |
| 206 | hCoV-19/South Korea/GJ-HERI-A0340/2021 | EPI_ISL_17702388 | AY.122 | 6-80 |
| 207 | hCoV-19/South Korea/GJ-HERI-A0341/2021 | EPI_ISL_17702389 | AY.122 | 6-81 |
| 208 | hCoV-19/South Korea/GJ-HERI-A0342/2021 | EPI_ISL_17702390 | AY.122 | 6-82 |
| 209 | hCoV-19/South Korea/GJ-HERI-A0343/2021 | EPI_ISL_17702391 | AY.122 | 6-83 |
| 210 | hCoV-19/South Korea/GJ-HERI-A0344/2021 | EPI_ISL_17702392 | AY.122 | 6-84 |
| 211 | hCoV-19/South Korea/GJ-HERI-A0345/2021 | EPI_ISL_17702393 | AY.69 | 6-85 |
| 212 | hCoV-19/South Korea/GJ-HERI-A0346/2021 | EPI_ISL_17702394 | AY.69 | 6-86 |
| 213 | hCoV-19/South Korea/GJ-HERI-A0347/2021 | EPI_ISL_17702395 | AY.69 | 6-87 |
| 214 | hCoV-19/South Korea/GJ-HERI-A0348/2021 | EPI_ISL_17702396 | AY.69 | 6-88 |
| 215 | hCoV-19/South Korea/GJ-HERI-A0349/2021 | EPI_ISL_17702397 | AY.122 | 6-89 |
| 216 | hCoV-19/South Korea/GJ-HERI-A0350/2021 | EPI_ISL_17702398 | AY.122 | 6-90 |
| 217 | hCoV-19/South Korea/GJ-HERI-A0351/2021 | EPI_ISL_17702399 | AY.122 | 6-91 |
| 218 | hCoV-19/South Korea/GJ-HERI-A0352/2021 | EPI_ISL_17702400 | AY.69 | 6-92 |
| 219 | hCoV-19/South Korea/GJ-HERI-A0353/2021 | EPI_ISL_17702401 | AY.69 | 6-93 |
| 220 | hCoV-19/South Korea/GJ-HERI-A0354/2021 | EPI_ISL_17702402 | AY.69 | 6-94 |
| 221 | hCoV-19/South Korea/GJ-HERI-A0355/2021 | EPI_ISL_17702403 | AY.69 | 6-95 |
| 222 | hCoV-19/South Korea/GJ-HERI-A0356/2021 | EPI_ISL_17702404 | AY.69 | 6-96 |
| 223 | hCoV-19/South Korea/GJ-HERI-A0357/2021 | EPI_ISL_17702405 | AY.69 | 6-97 |
| 224 | hCoV-19/South Korea/GJ-HERI-A0358/2021 | EPI_ISL_17702406 | AY.69 | 6-98 |
| 225 | hCoV-19/South Korea/GJ-HERI-A0359/2021 | EPI_ISL_17702407 | AY.122.5 | 6-99 |
| 226 | hCoV-19/South Korea/GJ-HERI-A0360/2021 | EPI_ISL_17702408 | AY.69 | 6-100 |
| 227 | hCoV-19/South Korea/GJ-HERI-A0361/2021 | EPI_ISL_17702409 | AY.69 | 6-101 |
| 228 | hCoV-19/South Korea/GJ-HERI-A0362/2021 | EPI_ISL_17702410 | AY.69 | 6-102 |
| 229 | hCoV-19/South Korea/GJ-HERI-A0363/2021 | EPI_ISL_17702411 | AY.69 | 6-103 |
| 230 | hCoV-19/South Korea/GJ-HERI-A0364/2021 | EPI_ISL_17702412 | AY.69 | 6-104 |
| 231 | hCoV-19/South Korea/GJ-HERI-A0376/2021 | EPI_ISL_17702424 | AY.69 | 6-105 |
| 232 | hCoV-19/South Korea/GJ-HERI-A0377/2021 | EPI_ISL_17702425 | AY.69 | 6-106 |
| 233 | hCoV-19/South Korea/GJ-HERI-A0378/2021 | EPI_ISL_17702426 | AY.69 | 6-107 |
| 234 | hCoV-19/South Korea/GJ-HERI-A0380/2021 | EPI_ISL_17702428 | AY.69 | 6-108 |
| 235 | hCoV-19/South Korea/GJ-HERI-A0387/2021 | EPI_ISL_17702435 | AY.69 | 6-109 |
| 236 | hCoV-19/South Korea/GJ-HERI-A0393/2021 | EPI_ISL_17702441 | AY.69 | 6-110 |
| 237 | hCoV-19/South Korea/GJ-HERI-A0394/2021 | EPI_ISL_17702442 | AY.69 | 6-111 |
| 238 | hCoV-19/South Korea/GJ-HERI-A0395/2021 | EPI_ISL_17702443 | AY.69 | 6-112 |
| 239 | hCoV-19/South Korea/GJ-HERI-A0398/2021 | EPI_ISL_17702446 | AY.69 | 6-113 |
| 240 | hCoV-19/South Korea/GJ-HERI-A0403/2021 | EPI_ISL_17702451 | AY.69 | 6-114 |
| 241 | hCoV-19/South Korea/GJ-HERI-A0404/2021 | EPI_ISL_17702452 | AY.69 | 6-115 |
| 242 | hCoV-19/South Korea/GJ-HERI-A0405/2021 | EPI_ISL_17702453 | AY.69 | 6-116 |
| 243 | hCoV-19/South Korea/GJ-HERI-A0406/2021 | EPI_ISL_17702454 | AY.69 | 6-117 |
| 244 | hCoV-19/South Korea/GJ-HERI-A0407/2021 | EPI_ISL_17702455 | AY.69 | 6-118 |
| 245 | hCoV-19/South Korea/GJ-HERI-A0401/2021 | EPI_ISL_17702449 | AY.69 | 6-119 |
| 246 | hCoV-19/South Korea/GJ-HERI-A0429/2022 | EPI_ISL_17702479 | BA.1.1 | 7-1 |
| 247 | hCoV-19/South Korea/GJ-HERI-A0434/2022 | EPI_ISL_17702484 | BA.1.1 | 7-2 |
| 248 | hCoV-19/South Korea/GJ-HERI-A0437/2022 | EPI_ISL_17702487 | BA.1.1 | 7-3 |
| 249 | hCoV-19/South Korea/GJ-HERI-A0438/2022 | EPI_ISL_17702488 | BA.1.1.5 | 7-4 |
| 250 | hCoV-19/South Korea/GJ-HERI-A0439/2022 | EPI_ISL_17702489 | BA.1.1 | 7-5 |
| 251 | hCoV-19/South Korea/GJ-HERI-A0440/2022 | EPI_ISL_17702490 | BA.1.1.5 | 7-6 |
| 252 | hCoV-19/South Korea/GJ-HERI-A0441/2022 | EPI_ISL_17702491 | BA.1.1 | 7-7 |
| 253 | hCoV-19/South Korea/GJ-HERI-A0442/2022 | EPI_ISL_17702492 | BA.1.1.5 | 7-8 |
| 254 | hCoV-19/South Korea/GJ-HERI-A0443/2022 | EPI_ISL_17702493 | BA.1.1.5 | 7-9 |
| 255 | hCoV-19/South Korea/GJ-HERI-A0444/2022 | EPI_ISL_17702494 | BA.1.1 | 7-10 |
| 256 | hCoV-19/South Korea/GJ-HERI-A0445/2022 | EPI_ISL_17702495 | BA.1.1.5 | 7-11 |
| 257 | hCoV-19/South Korea/GJ-HERI-A0446/2022 | EPI_ISL_17702496 | BA.1.1 | 7-12 |
| 258 | hCoV-19/South Korea/GJ-HERI-A0447/2022 | EPI_ISL_17702497 | BA.1.1 | 7-13 |
| 259 | hCoV-19/South Korea/GJ-HERI-A0448/2022 | EPI_ISL_17702498 | BA.1.1 | 7-14 |
| 260 | hCoV-19/South Korea/GJ-HERI-A0449/2022 | EPI_ISL_17702499 | BA.1.1 | 7-15 |
| 261 | hCoV-19/South Korea/GJ-HERI-A0450/2022 | EPI_ISL_17702500 | BA.1.1 | 7-16 |
| 262 | hCoV-19/South Korea/GJ-HERI-A0451/2022 | EPI_ISL_17702501 | BA.1.1 | 7-17 |
| 263 | hCoV-19/South Korea/GJ-HERI-A0459/2022 | EPI_ISL_17702509 | BA.1.1 | 7-18 |
| 264 | hCoV-19/South Korea/GJ-HERI-A0460/2022 | EPI_ISL_17702510 | BA.1.1 | 7-19 |
| 265 | hCoV-19/South Korea/GJ-HERI-A0461/2022 | EPI_ISL_17702511 | BA.1.1.5 | 7-20 |
| 266 | hCoV-19/South Korea/GJ-HERI-A0462/2022 | EPI_ISL_17702512 | BA.1.1.5 | 7-21 |
| 267 | hCoV-19/South Korea/GJ-HERI-A0476/2022 | EPI_ISL_17702526 | BA.1.1 | 7-22 |
| 268 | hCoV-19/South Korea/GJ-HERI-A0477/2022 | EPI_ISL_17702527 | BA.1.1.5 | 7-23 |
| 269 | hCoV-19/South Korea/GJ-HERI-A0478/2022 | EPI_ISL_17702528 | BA.1.1.5 | 7-24 |
| 270 | hCoV-19/South Korea/GJ-HERI-A0480/2022 | EPI_ISL_17702530 | BA.1.1 | 7-25 |
| 271 | hCoV-19/South Korea/GJ-HERI-A0481/2022 | EPI_ISL_17702531 | BA.1.1 | 7-26 |
| 272 | hCoV-19/South Korea/GJ-HERI-A0482/2022 | EPI_ISL_17702532 | BA.1.1 | 7-27 |
| 273 | hCoV-19/South Korea/GJ-HERI-A0483/2022 | EPI_ISL_17702533 | BA.1.1.5 | 7-28 |
| 274 | hCoV-19/South Korea/GJ-HERI-A0484/2022 | EPI_ISL_17702534 | BA.1.1 | 7-29 |
| 275 | hCoV-19/South Korea/GJ-HERI-A0485/2022 | EPI_ISL_17702535 | BA.1.1.5 | 7-30 |
| 276 | hCoV-19/South Korea/GJ-HERI-A0535/2022 | EPI_ISL_17702582 | BA.1.1.5 | 7-31 |
| 277 | hCoV-19/South Korea/GJ-HERI-A0536/2022 | EPI_ISL_17702583 | BA.1.1 | 7-32 |
| 278 | hCoV-19/South Korea/GJ-HERI-A0537/2022 | EPI_ISL_17702584 | BA.1.1 | 7-33 |
| 279 | hCoV-19/South Korea/GJ-HERI-A0538/2022 | EPI_ISL_17702585 | BA.1.1.5 | 7-34 |
| 280 | hCoV-19/South Korea/GJ-HERI-A0539/2022 | EPI_ISL_17702586 | BA.1.1.5 | 7-35 |
| 281 | hCoV-19/South Korea/GJ-HERI-A0540/2022 | EPI_ISL_17702587 | BA.1.1.5 | 7-36 |
| 282 | hCoV-19/South Korea/GJ-HERI-A0541/2022 | EPI_ISL_17702588 | BA.1.1.5 | 7-37 |
| 283 | hCoV-19/South Korea/GJ-HERI-A0542/2022 | EPI_ISL_17702589 | BA.1.1 | 7-38 |
| 284 | hCoV-19/South Korea/GJ-HERI-A0543/2022 | EPI_ISL_17702590 | BA.1.1 | 7-39 |
| 285 | hCoV-19/South Korea/GJ-HERI-A0544/2022 | EPI_ISL_17702591 | BA.1.1 | 7-40 |
| 286 | hCoV-19/South Korea/GJ-HERI-A0587/2022 | EPI_ISL_17702634 | BA.1.1 | 7-41 |
| 287 | hCoV-19/South Korea/GJ-HERI-A0588/2022 | EPI_ISL_17719137 | BA.1.1 | 7-42 |
| 288 | hCoV-19/South Korea/GJ-HERI-A0589/2022 | EPI_ISL_17702635 | BA.1.1 | 7-43 |
| 289 | hCoV-19/South Korea/GJ-HERI-A0590/2022 | EPI_ISL_17702636 | BA.1.1.5 | 7-44 |
| 290 | hCoV-19/South Korea/GJ-HERI-A0591/2022 | EPI_ISL_17702637 | BA.1.1.5 | 7-45 |
| 291 | hCoV-19/South Korea/GJ-HERI-A0592/2022 | EPI_ISL_17702638 | BA.1.1.5 | 7-46 |
| 292 | hCoV-19/South Korea/GJ-HERI-A0593/2022 | EPI_ISL_17702639 | BA.1.1.5 | 7-47 |
| 293 | hCoV-19/South Korea/GJ-HERI-A0594/2022 | EPI_ISL_17702640 | BA.1.1.5 | 7-48 |
| 294 | hCoV-19/South Korea/GJ-HERI-A0595/2022 | EPI_ISL_17702641 | BA.1.1 | 7-49 |
| 295 | hCoV-19/South Korea/GJ-HERI-A0596/2022 | EPI_ISL_17702642 | BA.1.1 | 7-50 |
| 296 | hCoV-19/South Korea/GJ-HERI-A0621/2022 | EPI_ISL_17702667 | BA.1.1.5 | 7-51 |
| 297 | hCoV-19/South Korea/GJ-HERI-A0622/2022 | EPI_ISL_17702668 | BA.1.1.5 | 7-52 |
| 298 | hCoV-19/South Korea/GJ-HERI-A0623/2022 | EPI_ISL_17702669 | BA.1.1 | 7-53 |
| 299 | hCoV-19/South Korea/GJ-HERI-A0624/2022 | EPI_ISL_17702670 | BA.1.1 | 7-54 |
| 300 | hCoV-19/South Korea/GJ-HERI-A0625/2022 | EPI_ISL_17702671 | BA.1.1.5 | 7-55 |
| 301 | hCoV-19/South Korea/GJ-HERI-A0626/2022 | EPI_ISL_17702672 | BA.1.1.5 | 7-56 |
| 302 | hCoV-19/South Korea/GJ-HERI-A0627/2022 | EPI_ISL_17702673 | BA.1.1 | 7-57 |
| 303 | hCoV-19/South Korea/GJ-HERI-A0630/2022 | EPI_ISL_17702676 | BA.1.1.5 | 7-58 |
| 304 | hCoV-19/South Korea/GJ-HERI-A0631/2022 | EPI_ISL_17702677 | BA.1.1 | 7-59 |
| 305 | hCoV-19/South Korea/GJ-HERI-A0632/2022 | EPI_ISL_17702678 | BA.1.1 | 7-60 |
| 306 | hCoV-19/South Korea/GJ-HERI-A0633/2022 | EPI_ISL_17702679 | BA.1.1.5 | 7-61 |
| 307 | hCoV-19/South Korea/GJ-HERI-A0634/2022 | EPI_ISL_17702680 | BA.1.1 | 7-62 |
| 308 | hCoV-19/South Korea/GJ-HERI-A0635/2022 | EPI_ISL_17702681 | BA.1.1.5 | 7-63 |
| 309 | hCoV-19/South Korea/GJ-HERI-A0636/2022 | EPI_ISL_17702682 | BA.1.1 | 7-64 |
| 310 | hCoV-19/South Korea/GJ-HERI-A0637/2022 | EPI_ISL_17702683 | BA.1.1.5 | 7-65 |
| 311 | hCoV-19/South Korea/GJ-HERI-A0638/2022 | EPI_ISL_17702684 | BA.1.17.2 | 7-66 |
| 312 | hCoV-19/South Korea/GJ-HERI-A0671/2022 | EPI_ISL_17702717 | BA.1.1.5 | 7-67 |
| 313 | hCoV-19/South Korea/GJ-HERI-A0672/2022 | EPI_ISL_17702718 | BA.1.1 | 7-68 |
| 314 | hCoV-19/South Korea/GJ-HERI-A0673/2022 | EPI_ISL_17702719 | BA.1.1 | 7-69 |
| 315 | hCoV-19/South Korea/GJ-HERI-A0674/2022 | EPI_ISL_17702720 | BA.1.1 | 7-70 |
| 316 | hCoV-19/South Korea/GJ-HERI-A0675/2022 | EPI_ISL_17702721 | BA.1.1.5 | 7-71 |
| 317 | hCoV-19/South Korea/GJ-HERI-A0676/2022 | EPI_ISL_17702722 | BA.1.1 | 7-72 |
| 318 | hCoV-19/South Korea/GJ-HERI-A0677/2022 | EPI_ISL_17702723 | BA.1.1 | 7-73 |
| 319 | hCoV-19/South Korea/GJ-HERI-A0678/2022 | EPI_ISL_17702724 | BA.1.1 | 7-74 |
| 320 | hCoV-19/South Korea/GJ-HERI-A0679/2022 | EPI_ISL_17702725 | BA.1.1 | 7-75 |
| 321 | hCoV-19/South Korea/GJ-HERI-A0680/2022 | EPI_ISL_17702726 | BA.1.1.5 | 7-76 |
| 322 | hCoV-19/South Korea/GJ-HERI-A0681/2022 | EPI_ISL_17702727 | BA.1.1 | 7-77 |
| 323 | hCoV-19/South Korea/GJ-HERI-A0689/2022 | EPI_ISL_17702768 | BA.2 | 8-1 |
| 324 | hCoV-19/South Korea/GJ-HERI-A0692/2022 | EPI_ISL_17702771 | BA.2.10 | 8-2 |
| 325 | hCoV-19/South Korea/GJ-HERI-A0693/2022 | EPI_ISL_17702772 | BA.2.3.13 | 8-3 |
| 326 | hCoV-19/South Korea/GJ-HERI-A0694/2022 | EPI_ISL_17702773 | BA.2.10 | 8-4 |
| 327 | hCoV-19/South Korea/GJ-HERI-A0695/2022 | EPI_ISL_17702774 | BA.2.3 | 8-5 |
| 328 | hCoV-19/South Korea/GJ-HERI-A0700/2022 | EPI_ISL_17702779 | BA.2.10 | 8-6 |
| 229 | hCoV-19/South Korea/GJ-HERI-A0701/2022 | EPI_ISL_17702780 | BA.2.10 | 8-7 |
| 330 | hCoV-19/South Korea/GJ-HERI-A0702/2022 | EPI_ISL_17702781 | BA.2.3 | 8-8 |
| 331 | hCoV-19/South Korea/GJ-HERI-A0703/2022 | EPI_ISL_17702782 | BA.2 | 8-9 |
| 332 | hCoV-19/South Korea/GJ-HERI-A0704/2022 | EPI_ISL_17702783 | BA.2.10 | 8-10 |
| 333 | hCoV-19/South Korea/GJ-HERI-A0705/2022 | EPI_ISL_17702784 | BA.2 | 8-11 |
| 334 | hCoV-19/South Korea/GJ-HERI-A0706/2022 | EPI_ISL_17702785 | BA.2.3 | 8-12 |
| 335 | hCoV-19/South Korea/GJ-HERI-A0707/2022 | EPI_ISL_17702786 | BA.2.10 | 8-13 |
| 336 | hCoV-19/South Korea/GJ-HERI-A0708/2022 | EPI_ISL_17702787 | BA.2.68 | 8-14 |
| 337 | hCoV-19/South Korea/GJ-HERI-A0727/2022 | EPI_ISL_17702805 | BA.2 | 8-15 |
| 338 | hCoV-19/South Korea/GJ-HERI-A0728/2022 | EPI_ISL_17702806 | BA.2.3 | 8-16 |
| 339 | hCoV-19/South Korea/GJ-HERI-A0730/2022 | EPI_ISL_17702808 | BA.2.3 | 8-17 |
| 340 | hCoV-19/South Korea/GJ-HERI-A0731/2022 | EPI_ISL_17702809 | BA.2.3 | 8-18 |
| 341 | hCoV-19/South Korea/GJ-HERI-A0732/2022 | EPI_ISL_17702810 | BA.2.3 | 8-19 |
| 342 | hCoV-19/South Korea/GJ-HERI-A0738/2022 | EPI_ISL_17702815 | BA.2.3 | 8-20 |
| 343 | hCoV-19/South Korea/GJ-HERI-A0739/2022 | EPI_ISL_17702816 | BA.2.3.12 | 8-21 |
| 344 | hCoV-19/South Korea/GJ-HERI-A0740/2022 | EPI_ISL_17702817 | BA.2.3 | 8-22 |
| 345 | hCoV-19/South Korea/GJ-HERI-A0747/2022 | EPI_ISL_17702824 | BA.2.3 | 8-23 |
| 346 | hCoV-19/South Korea/GJ-HERI-A0748/2022 | EPI_ISL_17702825 | BA.2.3 | 8-24 |
| 347 | hCoV-19/South Korea/GJ-HERI-A0749/2022 | EPI_ISL_17702826 | BA.2.65 | 8-25 |
| 348 | hCoV-19/South Korea/GJ-HERI-A0754/2022 | EPI_ISL_17702831 | BA.2.3 | 8-26 |
| 349 | hCoV-19/South Korea/GJ-HERI-A0755/2022 | EPI_ISL_17702832 | BA.2.68 | 8-27 |
| 350 | hCoV-19/South Korea/GJ-HERI-A0756/2022 | EPI_ISL_17702833 | BA.2.3.13 | 8-28 |
| 351 | hCoV-19/South Korea/GJ-HERI-A0757/2022 | EPI_ISL_17702834 | BA.2.3 | 8-29 |
| 352 | hCoV-19/South Korea/GJ-HERI-A0758/2022 | EPI_ISL_17702835 | BA.2.3 | 8-30 |
| 353 | hCoV-19/South Korea/GJ-HERI-A0868/2022 | EPI_ISL_17702945 | BA.2.3 | 8-31 |
| 354 | hCoV-19/South Korea/GJ-HERI-A0869/2022 | EPI_ISL_17702946 | BA.2.3 | 8-32 |
| 355 | hCoV-19/South Korea/GJ-HERI-A0870/2022 | EPI_ISL_17702947 | BA.2.3 | 8-33 |
| 356 | hCoV-19/South Korea/GJ-HERI-A0871/2022 | EPI_ISL_17702948 | BA.2.65 | 8-34 |
| 357 | hCoV-19/South Korea/GJ-HERI-A0872/2022 | EPI_ISL_17702949 | BA.2.3 | 8-35 |
| 358 | hCoV-19/South Korea/GJ-HERI-A0946/2022 | EPI_ISL_17703023 | BA.2.3 | 8-36 |
| 359 | hCoV-19/South Korea/GJ-HERI-A0947/2022 | EPI_ISL_17703024 | BA.2.3 | 8-37 |
| 360 | hCoV-19/South Korea/GJ-HERI-A0948/2022 | EPI_ISL_17703025 | BA.2.3.8 | 8-38 |
| 361 | hCoV-19/South Korea/GJ-HERI-A0949/2022 | EPI_ISL_17703026 | BA.2.3 | 8-39 |
| 362 | hCoV-19/South Korea/GJ-HERI-A0950/2022 | EPI_ISL_17703027 | BA.2.3 | 8-40 |
| 363 | hCoV-19/South Korea/GJ-HERI-A0976/2022 | EPI_ISL_17703053 | BA.2.3 | 8-41 |
| 364 | hCoV-19/South Korea/GJ-HERI-A0977/2022 | EPI_ISL_17703054 | BA.2.3 | 8-42 |
| 365 | hCoV-19/South Korea/GJ-HERI-A0978/2022 | EPI_ISL_17703055 | BA.2.3 | 8-43 |
| 366 | hCoV-19/South Korea/GJ-HERI-A0979/2022 | EPI_ISL_17703056 | BA.2.3 | 8-44 |
| 367 | hCoV-19/South Korea/GJ-HERI-A0980/2022 | EPI_ISL_17703057 | BA.2.65 | 8-45 |
| 368 | hCoV-19/South Korea/GJ-HERI-A1000/2022 | EPI_ISL_17703077 | BA.2 | 8-46 |
| 369 | hCoV-19/South Korea/GJ-HERI-A1001/2022 | EPI_ISL_17703078 | BA.2 | 8-47 |
| 370 | hCoV-19/South Korea/GJ-HERI-A1002/2022 | EPI_ISL_17703079 | BA.2.3 | 8-48 |
| 371 | hCoV-19/South Korea/GJ-HERI-A1003/2022 | EPI_ISL_17703080 | BA.2.3 | 8-49 |
| 372 | hCoV-19/South Korea/GJ-HERI-A1004/2022 | EPI_ISL_17703081 | BA.2 | 8-50 |
| 373 | hCoV-19/South Korea/GJ-HERI-A1044/2022 | EPI_ISL_17703123 | BA.2.3 | 8-51 |
| 374 | hCoV-19/South Korea/GJ-HERI-A1045/2022 | EPI_ISL_17703124 | BA.2.12.1 | 8-52 |
| 375 | hCoV-19/South Korea/GJ-HERI-A1046/2022 | EPI_ISL_17703125 | BA.2.65 | 8-53 |
| 376 | hCoV-19/South Korea/GJ-HERI-A1047/2022 | EPI_ISL_17703126 | BA.2.3 | 8-54 |
| 377 | hCoV-19/South Korea/GJ-HERI-A1048/2022 | EPI_ISL_17703127 | BA.2 | 8-55 |
| 378 | hCoV-19/South Korea/GJ-HERI-A1071/2022 | EPI_ISL_17703150 | BA.2.56 | 8-56 |
| 379 | hCoV-19/South Korea/GJ-HERI-A1074/2022 | EPI_ISL_17703153 | BA.2.12.1 | 8-57 |
| 380 | hCoV-19/South Korea/GJ-HERI-A1075/2022 | EPI_ISL_17703154 | BA.2.68 | 8-58 |
| 381 | hCoV-19/South Korea/GJ-HERI-A1088/2022 | EPI_ISL_17703167 | BA.2.12.1 | 8-59 |
| 382 | hCoV-19/South Korea/GJ-HERI-A1089/2022 | EPI_ISL_17703168 | BA.2.40.1 | 8-60 |
| 383 | hCoV-19/South Korea/GJ-HERI-A1134/2022 | EPI_ISL_17703213 | BA.2.3.12 | 8-61 |
| 384 | hCoV-19/South Korea/GJ-HERI-A1135/2022 | EPI_ISL_17703214 | BA.2.3 | 8-62 |
| 385 | hCoV-19/South Korea/GJ-HERI-A1136/2022 | EPI_ISL_17703215 | BA.2.3 | 8-63 |
| 386 | hCoV-19/South Korea/GJ-HERI-A1137/2022 | EPI_ISL_17703216 | BA.2.68 | 8-64 |
| 387 | hCoV-19/South Korea/GJ-HERI-A1138/2022 | EPI_ISL_17703217 | BA.2 | 8-65 |
| 388 | hCoV-19/South Korea/GJ-HERI-A1139/2022 | EPI_ISL_17703218 | BA.2.3 | 8-66 |
| 389 | hCoV-19/South Korea/GJ-HERI-A1140/2022 | EPI_ISL_17703219 | BA.2.3 | 8-67 |
| 390 | hCoV-19/South Korea/GJ-HERI-A1192/2022 | EPI_ISL_17703271 | BA.2 | 8-68 |
| 391 | hCoV-19/South Korea/GJ-HERI-A1193/2022 | EPI_ISL_17703272 | BA.2.3 | 8-69 |
| 392 | hCoV-19/South Korea/GJ-HERI-A1194/2022 | EPI_ISL_17703273 | BA.2.56 | 8-70 |
| 393 | hCoV-19/South Korea/GJ-HERI-A1195/2022 | EPI_ISL_17703274 | BA.2.3 | 8-71 |
| 394 | hCoV-19/South Korea/GJ-HERI-A1199/2022 | EPI_ISL_17703278 | BA.2.3 | 8-72 |
| 395 | hCoV-19/South Korea/GJ-HERI-A1200/2022 | EPI_ISL_17703279 | BA.2.65 | 8-73 |
| 396 | hCoV-19/South Korea/GJ-HERI-A1201/2022 | EPI_ISL_17703280 | BA.2.3 | 8-74 |
| 397 | hCoV-19/South Korea/GJ-HERI-A1202/2022 | EPI_ISL_17703281 | BA.2.3 | 8-75 |
| 398 | hCoV-19/South Korea/GJ-HERI-A1203/2022 | EPI_ISL_17703282 | BA.2.3 | 8-76 |
| 399 | hCoV-19/South Korea/GJ-HERI-A1204/2022 | EPI_ISL_17703283 | BA.2.3 | 8-77 |
| 400 | hCoV-19/South Korea/GJ-HERI-A1205/2022 | EPI_ISL_17703284 | BA.2.68 | 8-78 |
| 401 | hCoV-19/South Korea/GJ-HERI-A1206/2022 | EPI_ISL_17703285 | BA.2 | 8-79 |
| 402 | hCoV-19/South Korea/GJ-HERI-A1207/2022 | EPI_ISL_17703286 | BA.2.3.12 | 8-80 |
| 403 | hCoV-19/South Korea/GJ-HERI-A1208/2022 | EPI_ISL_17703287 | BA.2 | 8-81 |
| 404 | hCoV-19/South Korea/GJ-HERI-A1209/2022 | EPI_ISL_17703288 | BA.2.3.12 | 8-82 |
| 405 | hCoV-19/South Korea/GJ-HERI-A1210/2022 | EPI_ISL_17703289 | BA.2 | 8-83 |
| 406 | hCoV-19/South Korea/GJ-HERI-A1228/2022 | EPI_ISL_17703307 | BA.2.74 | 8-84 |
| 407 | hCoV-19/South Korea/GJ-HERI-A1229/2022 | EPI_ISL_17703308 | BA.2 | 8-85 |
| 408 | hCoV-19/South Korea/GJ-HERI-A1230/2022 | EPI_ISL_17703309 | BA.2.3.12 | 8-86 |
| 409 | hCoV-19/South Korea/GJ-HERI-A1231/2022 | EPI_ISL_17703310 | BA.2.65 | 8-87 |
| 410 | hCoV-19/South Korea/GJ-HERI-A1232/2022 | EPI_ISL_17703311 | BA.2.65 | 8-88 |
| 411 | hCoV-19/South Korea/GJ-HERI-A1236/2022 | EPI_ISL_17703315 | BA.2.75.2 | 8-89 |
| 412 | hCoV-19/South Korea/GJ-HERI-A1237/2022 | EPI_ISL_17703316 | BA.2 | 8-90 |
| 413 | hCoV-19/South Korea/GJ-HERI-A1238/2022 | EPI_ISL_17703317 | BA.2 | 8-91 |
| 414 | hCoV-19/South Korea/GJ-HERI-A1239/2022 | EPI_ISL_17703318 | BA.2 | 8-92 |
| 415 | hCoV-19/South Korea/GJ-HERI-A1240/2022 | EPI_ISL_17703319 | BA.2.38.1 | 8-93 |
| 416 | hCoV-19/South Korea/GJ-HERI-A1244/2022 | EPI_ISL_17703323 | BA.2.3.7 | 8-94 |
| 417 | hCoV-19/South Korea/GJ-HERI-A1245/2022 | EPI_ISL_17703324 | BA.2.56 | 8-95 |
| 418 | hCoV-19/South Korea/GJ-HERI-A1246/2022 | EPI_ISL_17703325 | BA.2.75.2 | 8-96 |
| 419 | hCoV-19/South Korea/GJ-HERI-A1251/2022 | EPI_ISL_17703330 | BA.2.3.20 | 8-97 |
| 420 | hCoV-19/South Korea/GJ-HERI-A1254/2022 | EPI_ISL_17703333 | BA.2.75.3 | 8-98 |
| 421 | hCoV-19/South Korea/GJ-HERI-A1257/2022 | EPI_ISL_17703336 | BA.2.3.20 | 8-99 |
| 422 | hCoV-19/South Korea/GJ-HERI-A1258/2022 | EPI_ISL_17703337 | CM.1 | 8-100 |
| 423 | hCoV-19/South Korea/GJ-HERI-A1260/2022 | EPI_ISL_17703339 | BA.2.3.20 | 8-101 |
| 424 | hCoV-19/South Korea/GJ-HERI-A1269/2022 | EPI_ISL_17703348 | BL.1 | 8-102 |
| 425 | hCoV-19/South Korea/GJ-HERI-A1270/2022 | EPI_ISL_17703349 | BL.1 | 8-103 |
| 426 | hCoV-19/South Korea/GJ-HERI-A1276/2022 | EPI_ISL_17703355 | CH.1.1 | 8-104 |
| 427 | hCoV-19/South Korea/GJ-HERI-A1280/2022 | EPI_ISL_17703359 | CH.1.1 | 8-105 |
| 428 | hCoV-19/South Korea/GJ-HERI-A1281/2022 | EPI_ISL_17703360 | BA.2.3.20 | 8-106 |
| 429 | hCoV-19/South Korea/GJ-HERI-A1296/2022 | EPI_ISL_17703375 | BN.1.3 | 8-107 |
| 430 | hCoV-19/South Korea/GJ-HERI-A1297/2022 | EPI_ISL_17703376 | BN.1.3 | 8-108 |
| 431 | hCoV-19/South Korea/GJ-HERI-A1298/2022 | EPI_ISL_17703377 | BA.2.75.4 | 8-109 |
| 432 | hCoV-19/South Korea/GJ-HERI-A1299/2022 | EPI_ISL_17703378 | BN.1.3 | 8-110 |
| 433 | hCoV-19/South Korea/GJ-HERI-A1300/2022 | EPI_ISL_17703379 | BA.2.3.20 | 8-111 |
| 434 | hCoV-19/South Korea/GJ-HERI-A1345/2022 | EPI_ISL_17703424 | BN.1.2 | 8-112 |
| 435 | hCoV-19/South Korea/GJ-HERI-A1346/2022 | EPI_ISL_17703425 | BN.1.3 | 8-113 |
| 436 | hCoV-19/South Korea/GJ-HERI-A1347/2022 | EPI_ISL_17703426 | BL.1 | 8-114 |
| 437 | hCoV-19/South Korea/GJ-HERI-A1348/2022 | EPI_ISL_17703427 | CM.4 | 8-115 |
| 438 | hCoV-19/South Korea/GJ-HERI-A1363/2022 | EPI_ISL_17703442 | BN.1.2 | 8-116 |
| 439 | hCoV-19/South Korea/GJ-HERI-A1364/2022 | EPI_ISL_17703443 | BN.1.3 | 8-117 |
| 440 | hCoV-19/South Korea/GJ-HERI-A1365/2022 | EPI_ISL_17703444 | BN.1.2 | 8-118 |
| 441 | hCoV-19/South Korea/GJ-HERI-A1366/2022 | EPI_ISL_17703445 | BA.2.75.4 | 8-119 |
| 442 | hCoV-19/South Korea/GJ-HERI-A1367/2022 | EPI_ISL_17703446 | BN.4 | 8-120 |
| 443 | hCoV-19/South Korea/GJ-HERI-A1378/2022 | EPI_ISL_17703457 | BA.4.1.1 | 9-1 |
| 444 | hCoV-19/South Korea/GJ-HERI-A1379/2022 | EPI_ISL_17703458 | BA.4.1.1 | 9-2 |
| 445 | hCoV-19/South Korea/GJ-HERI-A1382/2022 | EPI_ISL_17703461 | BA.4.1.1 | 9-3 |
| 446 | hCoV-19/South Korea/GJ-HERI-A1383/2022 | EPI_ISL_17703462 | BA.4.1.1 | 9-4 |
| 447 | hCoV-19/South Korea/GJ-HERI-A1384/2022 | EPI_ISL_17703463 | BA.4.1 | 9-5 |
| 448 | hCoV-19/South Korea/GJ-HERI-A1389/2022 | EPI_ISL_17703468 | BA.4.4 | 9-6 |
| 449 | hCoV-19/South Korea/GJ-HERI-A1390/2022 | EPI_ISL_17719130 | BA.4.1.8 | 9-7 |
| 450 | hCoV-19/South Korea/GJ-HERI-A1391/2022 | EPI_ISL_17703469 | BA.4.1 | 9-8 |
| 451 | hCoV-19/South Korea/GJ-HERI-A1392/2022 | EPI_ISL_17703470 | BA.4.1 | 9-9 |
| 452 | hCoV-19/South Korea/GJ-HERI-A1408/2022 | EPI_ISL_17697006 | BA.5.1.10 | 10-1 |
| 453 | hCoV-19/South Korea/GJ-HERI-A1409/2022 | EPI_ISL_17697007 | BA.5.2.9 | 10-2 |
| 454 | hCoV-19/South Korea/GJ-HERI-A1410/2022 | EPI_ISL_17697008 | BA.5.2.9 | 10-3 |
| 455 | hCoV-19/South Korea/GJ-HERI-A1411/2022 | EPI_ISL_17697009 | BA.5.2.9 | 10-4 |
| 456 | hCoV-19/South Korea/GJ-HERI-A1412/2022 | EPI_ISL_17697010 | BF.21 | 10-5 |
| 457 | hCoV-19/South Korea/GJ-HERI-A1413/2022 | EPI_ISL_17697011 | BA.5.1.10 | 10-6 |
| 458 | hCoV-19/South Korea/GJ-HERI-A1416/2022 | EPI_ISL_17697014 | BA.5.2 | 10-7 |
| 459 | hCoV-19/South Korea/GJ-HERI-A1417/2022 | EPI_ISL_17697015 | BE.1.1 | 10-8 |
| 460 | hCoV-19/South Korea/GJ-HERI-A1418/2022 | EPI_ISL_17697016 | BA.5.2.1 | 10-9 |
| 461 | hCoV-19/South Korea/GJ-HERI-A1419/2022 | EPI_ISL_17697017 | BA.5.5 | 10-10 |
| 462 | hCoV-19/South Korea/GJ-HERI-A1420/2022 | EPI_ISL_17697018 | BA.5.5 | 10-11 |
| 463 | hCoV-19/South Korea/GJ-HERI-A1421/2022 | EPI_ISL_17697019 | BA.5.5 | 10-12 |
| 464 | hCoV-19/South Korea/GJ-HERI-A1422/2022 | EPI_ISL_17697020 | BA.5.2 | 10-13 |
| 465 | hCoV-19/South Korea/GJ-HERI-A1423/2022 | EPI_ISL_17697021 | BA.5.2 | 10-14 |
| 466 | hCoV-19/South Korea/GJ-HERI-A1454/2022 | EPI_ISL_17697052 | BA.5.2 | 10-15 |
| 467 | hCoV-19/South Korea/GJ-HERI-A1455/2022 | EPI_ISL_17697053 | BA.5.1 | 10-16 |
| 468 | hCoV-19/South Korea/GJ-HERI-A1456/2022 | EPI_ISL_17697054 | BA.5.2 | 10-17 |
| 469 | hCoV-19/South Korea/GJ-HERI-A1457/2022 | EPI_ISL_17697055 | BA.5.1 | 10-18 |
| 470 | hCoV-19/South Korea/GJ-HERI-A1458/2022 | EPI_ISL_17697056 | BA.5.2.1 | 10-19 |
| 471 | hCoV-19/South Korea/GJ-HERI-A1459/2022 | EPI_ISL_17697057 | BA.5.2 | 10-20 |
| 472 | hCoV-19/South Korea/GJ-HERI-A1460/2022 | EPI_ISL_17697058 | BA.5.2.1 | 10-21 |
| 473 | hCoV-19/South Korea/GJ-HERI-A1461/2022 | EPI_ISL_17697059 | BA.5.2.27 | 10-22 |
| 474 | hCoV-19/South Korea/GJ-HERI-A1462/2022 | EPI_ISL_17697060 | BA.5.10 | 10-23 |
| 475 | hCoV-19/South Korea/GJ-HERI-A1463/2022 | EPI_ISL_17697061 | BA.5.2 | 10-24 |
| 476 | hCoV-19/South Korea/GJ-HERI-A1464/2022 | EPI_ISL_17697062 | BA.5.2 | 10-25 |
| 477 | hCoV-19/South Korea/GJ-HERI-A1465/2022 | EPI_ISL_17697063 | BA.5.1 | 10-26 |
| 478 | hCoV-19/South Korea/GJ-HERI-A1466/2022 | EPI_ISL_17697064 | BA.5.1 | 10-27 |
| 479 | hCoV-19/South Korea/GJ-HERI-A1473/2022 | EPI_ISL_17697071 | BA.5.5 | 10-28 |
| 480 | hCoV-19/South Korea/GJ-HERI-A1474/2022 | EPI_ISL_17697072 | BA.5.2.1 | 10-29 |
| 481 | hCoV-19/South Korea/GJ-HERI-A1475/2022 | EPI_ISL_17697073 | BA.5.2 | 10-30 |
| 482 | hCoV-19/South Korea/GJ-HERI-A1476/2022 | EPI_ISL_17697074 | BA.5.5 | 10-31 |
| 483 | hCoV-19/South Korea/GJ-HERI-A1477/2022 | EPI_ISL_17697075 | BA.5.2.1 | 10-32 |
| 484 | hCoV-19/South Korea/GJ-HERI-A1478/2022 | EPI_ISL_17697076 | BA.5.2 | 10-33 |
| 485 | hCoV-19/South Korea/GJ-HERI-A1479/2022 | EPI_ISL_17697077 | BA.5.5 | 10-34 |
| 486 | hCoV-19/South Korea/GJ-HERI-A1489/2022 | EPI_ISL_17697087 | BA.5.2 | 10-35 |
| 487 | hCoV-19/South Korea/GJ-HERI-A1490/2022 | EPI_ISL_17697088 | BF.3 | 10-36 |
| 488 | hCoV-19/South Korea/GJ-HERI-A1491/2022 | EPI_ISL_17697089 | BA.5.8 | 10-37 |
| 489 | hCoV-19/South Korea/GJ-HERI-A1492/2022 | EPI_ISL_17697090 | BF.5 | 10-38 |
| 490 | hCoV-19/South Korea/GJ-HERI-A1493/2022 | EPI_ISL_17697091 | BF.5 | 10-39 |
| 491 | hCoV-19/South Korea/GJ-HERI-A1494/2022 | EPI_ISL_17697092 | BA.5.2 | 10-40 |
| 492 | hCoV-19/South Korea/GJ-HERI-A1508/2022 | EPI_ISL_17697106 | BA.5.1 | 10-41 |
| 493 | hCoV-19/South Korea/GJ-HERI-A1509/2022 | EPI_ISL_17697107 | BA.5.1 | 10-42 |
| 494 | hCoV-19/South Korea/GJ-HERI-A1510/2022 | EPI_ISL_17697108 | BA.5.2.20 | 10-43 |
| 495 | hCoV-19/South Korea/GJ-HERI-A1511/2022 | EPI_ISL_17697109 | BF.21 | 10-44 |
| 496 | hCoV-19/South Korea/GJ-HERI-A1512/2022 | EPI_ISL_17697110 | BA.5.2.1 | 10-45 |
| 497 | hCoV-19/South Korea/GJ-HERI-A1513/2022 | EPI_ISL_17697111 | BA.5.5 | 10-46 |
| 498 | hCoV-19/South Korea/GJ-HERI-A1514/2022 | EPI_ISL_17697112 | BF.10 | 10-47 |
| 499 | hCoV-19/South Korea/GJ-HERI-A1515/2022 | EPI_ISL_17697113 | BA.5.9 | 10-48 |
| 500 | hCoV-19/South Korea/GJ-HERI-A1516/2022 | EPI_ISL_17697114 | BA.5.6 | 10-49 |
| 501 | hCoV-19/South Korea/GJ-HERI-A1559/2022 | EPI_ISL_17697158 | BF.24 | 10-50 |
| 502 | hCoV-19/South Korea/GJ-HERI-A1560/2022 | EPI_ISL_17697159 | BA.5.2.3 | 10-51 |
| 503 | hCoV-19/South Korea/GJ-HERI-A1561/2022 | EPI_ISL_17697160 | BA.5.2 | 10-52 |
| 504 | hCoV-19/South Korea/GJ-HERI-A1562/2022 | EPI_ISL_17697161 | BA.5.2 | 10-53 |
| 505 | hCoV-19/South Korea/GJ-HERI-A1563/2022 | EPI_ISL_17697162 | BA.5.2 | 10-54 |
| 506 | hCoV-19/South Korea/GJ-HERI-A1564/2022 | EPI_ISL_17697163 | BA.5.1 | 10-55 |
| 507 | hCoV-19/South Korea/GJ-HERI-A1565/2022 | EPI_ISL_17697164 | BA.5.2.22 | 10-56 |
| 508 | hCoV-19/South Korea/GJ-HERI-A1566/2022 | EPI_ISL_17697165 | BA.5.5 | 10-57 |
| 509 | hCoV-19/South Korea/GJ-HERI-A1567/2022 | EPI_ISL_17697166 | BA.5.2 | 10-58 |
| 510 | hCoV-19/South Korea/GJ-HERI-A1568/2022 | EPI_ISL_17697167 | BA.5.2.1 | 10-59 |
| 511 | hCoV-19/South Korea/GJ-HERI-A1642/2022 | EPI_ISL_17697238 | BA.5.2.1 | 10-60 |
| 512 | hCoV-19/South Korea/GJ-HERI-A1643/2022 | EPI_ISL_17697239 | BA.5.2.9 | 10-61 |
| 513 | hCoV-19/South Korea/GJ-HERI-A1644/2022 | EPI_ISL_17697240 | BA.5.2 | 10-62 |
| 514 | hCoV-19/South Korea/GJ-HERI-A1645/2022 | EPI_ISL_17697241 | BF.27 | 10-63 |
| 515 | hCoV-19/South Korea/GJ-HERI-A1646/2022 | EPI_ISL_17697242 | BA.5.2.1 | 10-64 |
| 516 | hCoV-19/South Korea/GJ-HERI-A1647/2022 | EPI_ISL_17697243 | BA.5.6 | 10-65 |
| 517 | hCoV-19/South Korea/GJ-HERI-A1648/2022 | EPI_ISL_17697244 | BA.5.2.19 | 10-66 |
| 518 | hCoV-19/South Korea/GJ-HERI-A1649/2022 | EPI_ISL_17697245 | BA.5.2.1 | 10-67 |
| 519 | hCoV-19/South Korea/GJ-HERI-A1650/2022 | EPI_ISL_17697246 | BA.5.2 | 10-68 |
| 520 | hCoV-19/South Korea/GJ-HERI-A1651/2022 | EPI_ISL_17697247 | BA.5.1 | 10-69 |
| 521 | hCoV-19/South Korea/GJ-HERI-A1652/2022 | EPI_ISL_17697248 | BA.5.2 | 10-70 |
| 522 | hCoV-19/South Korea/GJ-HERI-A1817/2022 | EPI_ISL_17697414 | BA.5.2 | 10-71 |
| 523 | hCoV-19/South Korea/GJ-HERI-A1818/2022 | EPI_ISL_17697415 | BA.5.2.1 | 10-72 |
| 524 | hCoV-19/South Korea/GJ-HERI-A1819/2022 | EPI_ISL_17697416 | BA.5.1 | 10-73 |
| 525 | hCoV-19/South Korea/GJ-HERI-A1820/2022 | EPI_ISL_17697417 | BA.5.2 | 10-74 |
| 526 | hCoV-19/South Korea/GJ-HERI-A1821/2022 | EPI_ISL_17697418 | BF.5 | 10-75 |
| 527 | hCoV-19/South Korea/GJ-HERI-A1822/2022 | EPI_ISL_17697419 | BA.5.5 | 10-76 |
| 528 | hCoV-19/South Korea/GJ-HERI-A1823/2022 | EPI_ISL_17697420 | BA.5.2.1 | 10-77 |
| 529 | hCoV-19/South Korea/GJ-HERI-A1824/2022 | EPI_ISL_17697421 | BA.5.2.1 | 10-78 |
| 530 | hCoV-19/South Korea/GJ-HERI-A1825/2022 | EPI_ISL_17697422 | BA.5.2 | 10-79 |
| 531 | hCoV-19/South Korea/GJ-HERI-A1826/2022 | EPI_ISL_17697423 | BA.5.1 | 10-80 |
| 532 | hCoV-19/South Korea/GJ-HERI-A1962/2022 | EPI_ISL_17698348 | BF.21 | 10-81 |
| 533 | hCoV-19/South Korea/GJ-HERI-A1963/2022 | EPI_ISL_17698349 | BA.5.2.1 | 10-82 |
| 534 | hCoV-19/South Korea/GJ-HERI-A1964/2022 | EPI_ISL_17698350 | BE.1 | 10-83 |
| 535 | hCoV-19/South Korea/GJ-HERI-A1965/2022 | EPI_ISL_17698351 | BA.5.2 | 10-84 |
| 536 | hCoV-19/South Korea/GJ-HERI-A1966/2022 | EPI_ISL_17698352 | BA.5.2.16 | 10-85 |
| 537 | hCoV-19/South Korea/GJ-HERI-A1967/2022 | EPI_ISL_17698353 | BA.5.2 | 10-86 |
| 538 | hCoV-19/South Korea/GJ-HERI-A1968/2022 | EPI_ISL_17698354 | BF.5 | 10-87 |
| 539 | hCoV-19/South Korea/GJ-HERI-A1969/2022 | EPI_ISL_17698355 | BA.5.2.1 | 10-88 |
| 540 | hCoV-19/South Korea/GJ-HERI-A1970/2022 | EPI_ISL_17698356 | BA.5.2.1 | 10-89 |
| 541 | hCoV-19/South Korea/GJ-HERI-A2012/2022 | EPI_ISL_17698398 | BA.5.2 | 10-90 |
| 542 | hCoV-19/South Korea/GJ-HERI-A2013/2022 | EPI_ISL_17698399 | BA.5.2 | 10-91 |
| 543 | hCoV-19/South Korea/GJ-HERI-A2014/2022 | EPI_ISL_17698400 | BA.5.2.1 | 10-92 |
| 544 | hCoV-19/South Korea/GJ-HERI-A2015/2022 | EPI_ISL_17698401 | BQ.1.1 | 10-93 |
| 545 | hCoV-19/South Korea/GJ-HERI-A2016/2022 | EPI_ISL_17698402 | BA.5.2.19 | 10-94 |
| 546 | hCoV-19/South Korea/GJ-HERI-A2017/2022 | EPI_ISL_17698403 | BA.5.2 | 10-95 |
| 547 | hCoV-19/South Korea/GJ-HERI-A2018/2022 | EPI_ISL_17698404 | BA.5.2.1 | 10-96 |
| 548 | hCoV-19/South Korea/GJ-HERI-A2019/2022 | EPI_ISL_17698405 | BF.7 | 10-97 |
| 549 | hCoV-19/South Korea/GJ-HERI-A2020/2022 | EPI_ISL_17698406 | BA.5.1 | 10-98 |
| 550 | hCoV-19/South Korea/GJ-HERI-A2056/2022 | EPI_ISL_17698442 | BA.5.2.19 | 10-99 |
| 551 | hCoV-19/South Korea/GJ-HERI-A2057/2022 | EPI_ISL_17698443 | BA.5.2.22 | 10-100 |
| 552 | hCoV-19/South Korea/GJ-HERI-A2058/2022 | EPI_ISL_17698444 | BA.5.1 | 10-101 |
| 553 | hCoV-19/South Korea/GJ-HERI-A2059/2022 | EPI_ISL_17698445 | BF.5 | 10-102 |
| 554 | hCoV-19/South Korea/GJ-HERI-A2060/2022 | EPI_ISL_17698446 | BA.5.2 | 10-103 |
| 555 | hCoV-19/South Korea/GJ-HERI-A2061/2022 | EPI_ISL_17698447 | BA.5.2.1 | 10-104 |
| 556 | hCoV-19/South Korea/GJ-HERI-A2100/2022 | EPI_ISL_17698486 | BA.5.2.1 | 10-105 |
| 557 | hCoV-19/South Korea/GJ-HERI-A2101/2022 | EPI_ISL_17698487 | BA.5.2.27 | 10-106 |
| 558 | hCoV-19/South Korea/GJ-HERI-A2102/2022 | EPI_ISL_17698488 | BA.5.2 | 10-107 |
| 559 | hCoV-19/South Korea/GJ-HERI-A2113/2022 | EPI_ISL_17698498 | BA.5.2.1 | 10-108 |
| 560 | hCoV-19/South Korea/GJ-HERI-A2114/2022 | EPI_ISL_17698499 | BA.5.2.1 | 10-109 |
| 561 | hCoV-19/South Korea/GJ-HERI-A2115/2022 | EPI_ISL_17699376 | BQ.1.1.1 | 10-110 |
| 562 | hCoV-19/South Korea/GJ-HERI-A2116/2022 | EPI_ISL_17698500 | BA.5.2.1 | 10-111 |
| 563 | hCoV-19/South Korea/GJ-HERI-A2136/2022 | EPI_ISL_17698520 | BA.5.2.1 | 10-112 |
| 564 | hCoV-19/South Korea/GJ-HERI-A2137/2022 | EPI_ISL_17698521 | BA.5.2 | 10-113 |
| 565 | hCoV-19/South Korea/GJ-HERI-A2138/2022 | EPI_ISL_17698522 | BA.5.2.22 | 10-114 |
| 566 | hCoV-19/South Korea/GJ-HERI-A2139/2022 | EPI_ISL_17698523 | BA.5.2 | 10-115 |
| 567 | hCoV-19/South Korea/GJ-HERI-A2140/2022 | EPI_ISL_17698524 | BA.5.2 | 10-116 |
| 568 | hCoV-19/South Korea/GJ-HERI-A2141/2022 | EPI_ISL_17698525 | BA.5.2 | 10-117 |
| 569 | hCoV-19/South Korea/GJ-HERI-A2142/2022 | EPI_ISL_17698526 | BA.5.2 | 10-118 |
| 570 | hCoV-19/South Korea/GJ-HERI-A2157/2022 | EPI_ISL_17698541 | BA.5.2.1 | 10-119 |
| 571 | hCoV-19/South Korea/GJ-HERI-A2158/2022 | EPI_ISL_17698542 | BA.5.2 | 10-120 |
| 572 | hCoV-19/South Korea/GJ-HERI-A2159/2022 | EPI_ISL_17698543 | BA.5.2 | 10-121 |
| 573 | hCoV-19/South Korea/GJ-HERI-A2160/2022 | EPI_ISL_17698544 | BA.5.2 | 10-122 |
| 574 | hCoV-19/South Korea/GJ-HERI-A2161/2022 | EPI_ISL_17698545 | BA.5.2 | 10-123 |
| 575 | hCoV-19/South Korea/GJ-HERI-A2200/2022 | EPI_ISL_17698582 | BA.5.2.1 | 10-124 |
| 576 | hCoV-19/South Korea/GJ-HERI-A2201/2022 | EPI_ISL_17698583 | BQ.1.1 | 10-125 |
| 577 | hCoV-19/South Korea/GJ-HERI-A2202/2022 | EPI_ISL_17698584 | CM.4 | 10-126 |
| 578 | hCoV-19/South Korea/GJ-HERI-A2203/2022 | EPI_ISL_17698585 | CM.4 | 10-127 |
| 579 | hCoV-19/South Korea/GJ-HERI-A2204/2022 | EPI_ISL_17698586 | CM.8.1 | 10-128 |
| 580 | hCoV-19/South Korea/GJ-HERI-A2205/2022 | EPI_ISL_17698587 | BQ.1.1 | 10-129 |
| 581 | hCoV-19/South Korea/GJ-HERI-A2206/2022 | EPI_ISL_17698588 | BA.5.1.5 | 10-130 |
| 582 | hCoV-19/South Korea/GJ-HERI-A2207/2022 | EPI_ISL_17698589 | BA.5.2.1 | 10-131 |
| 583 | hCoV-19/South Korea/GJ-HERI-A2337/2022 | EPI_ISL_17698719 | BA.5.2 | 10-132 |
| 584 | hCoV-19/South Korea/GJ-HERI-A2338/2022 | EPI_ISL_17698720 | BA.5.2 | 10-133 |
| 585 | hCoV-19/South Korea/GJ-HERI-A2340/2022 | EPI_ISL_17698722 | BQ.1.11 | 10-134 |
| 586 | hCoV-19/South Korea/GJ-HERI-A2344/2022 | EPI_ISL_17698726 | BQ.1.3 | 10-135 |
| 587 | hCoV-19/South Korea/GJ-HERI-A2363/2022 | EPI_ISL_17698745 | BA.5.2.44 | 10-136 |
| 588 | hCoV-19/South Korea/GJ-HERI-A2364/2022 | EPI_ISL_17698746 | BA.5.2.1 | 10-137 |
| 589 | hCoV-19/South Korea/GJ-HERI-A2365/2022 | EPI_ISL_17698747 | BQ.1.2 | 10-138 |
| 590 | hCoV-19/South Korea/GJ-HERI-A2366/2022 | EPI_ISL_17698748 | BQ.1.1 | 10-139 |
| 591 | hCoV-19/South Korea/GJ-HERI-A2393/2022 | EPI_ISL_17698775 | BQ.1.1.17 | 10-140 |
| 592 | hCoV-19/South Korea/GJ-HERI-A2416/2022 | EPI_ISL_17698802 | XBB.1 | 11-1 |
| 593 | hCoV-19/South Korea/GJ-HERI-A2417/2022 | EPI_ISL_17698803 | XBB | 11-2 |
| 594 | hCoV-19/South Korea/GJ-HERI-A2418/2022 | EPI_ISL_17698804 | XBB.1 | 11-3 |
| 595 | hCoV-19/South Korea/GJ-HERI-A2420/2022 | EPI_ISL_17698806 | XBB.1 | 11-4 |
| 596 | hCoV-19/South Korea/GJ-HERI-A2423/2022 | EPI_ISL_17698809 | XBB.1 | 11-5 |

Supplementary Table 3. Table of 61 amino acid mutations identified in all 86 sequences of B.1.619.1

| Gene | Mutation Type (Amino acid change) | |
| --- | --- | --- |
|  | Common mutations | Different mutations |
| *ORF1ab* | A2123V, E2607K, S3675del, G3676del, F3677del, M3752I, K3929R, P4715L | P380S, F548S, T1597I, D1885Y, I2010M, H2092Y, T2152A, S2193F, M2259I, K2511N, T2967L, A3454T, L3829F, E4097G, V5422F, D5528Y, P5624L, L6102F, A6199S, V6579F, S6739L, T6854I |
| *S* | I210T, N440K, E484K, D614G, D936N,  S939F, T1027I | T20I, T1009I, D1199H, E1207D, C1236F |
| *ORF3a* | - | W69L, L85F, V256fs |
| *M* | I82T | - |
| *ORF7a* | E22D | V29L, P34L, G38V, G42S |
| *ORF8* | - | C25F, I121fs |
| *N* | P13L, S201I, T205I | S78D, T135I, A152S, P199S, L221F |
